# Real‐Time Detection of Hot Electrons During Chemical Reactions with Catalytic Nanodevices

**DOI:** 10.1002/advs.202512017

**Published:** 2025-08-11

**Authors:** Yeji Yoon, Si Woo Lee

**Affiliations:** ^1^ Department of Chemistry and Chemical Engineering Inha University Incheon 22212 Republic of Korea

**Keywords:** catalytic nanodiode, energy conversion, heterogeneous catalysis, hot electrons, schottky junction

## Abstract

Hot electrons generated via non‐adiabatic electronic excitations during catalytic reactions offer new insights into charge transfer dynamics at catalytic interfaces. Recent advancements in catalytic nanodevices, particularly metal–semiconductor Schottky nanodiodes, have enabled real‐time detection of hot electrons via chemicurrent measurements. Initial studies focused on thin‐film‐based nanodiodes under vacuum conditions, but more advanced studies conducted under ambient‐pressure environments reveal a direct correlation between hot electron excitation and catalytic performance. Furthermore, integrating nanocatalysts into nanodiodes has narrowed the gap between model systems and practical catalysts, demonstrating hot electron excitation in nanoparticle‐based systems. This review highlights key experimental developments in hot electron research, discussing strategies to enhance detection efficiency and potential applications in catalysis. The ability to manipulate electron flow at catalytic interfaces suggests future opportunities for electronically tunable catalysis, offering a pathway toward energy‐efficient and selective reaction control.

## Graphical abstract

This Review highlights recent advances in the detection of hot electrons generated during catalytic surface reactions through non‐adiabatic energy dissipation. By integrating metal–semiconductor junctions into catalytic nanodevices, researchers can monitor charge carrier flow in real time, offering new insights into interfacial energy conversion, surface reaction mechanisms, and the development of electronically responsive catalytic systems.

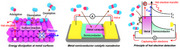

## 1 Introduction: Fundamentals of Generation, Transport, and Detection of Hot Electrons under Catalytic Reactions

Charge transfer at catalytic interfaces plays a crucial role in determining reaction rates, selectivity, and energy dissipation pathways.^[^ ^1^, ^2^ ^]^ In heterogeneous catalysis, chemical transformations involve dynamic electron exchange between reactants and catalyst surfaces, which influences reaction kinetics and product distributions.^[^ ^3^, ^4^, ^5^, ^6^, ^7^, ^8^, ^9^ ^]^ The extent and direction of charge transfer can alter activation barriers, stabilize reaction intermediates, and modulate catalytic activity.^[^ ^10^, ^11^, ^12^, ^13^ ^]^ Since electron transfer, while present, plays a limited and indirect role in most catalytic processes, real‐time detection of charge carriers during chemical reactions has emerged as a useful tool for probing nonadiabatic energy dissipation phenomena. However, direct observation of reaction‐induced electron flow remains a challenge due to the ultrafast timescales and short transport distances involved.^[^ ^14^, ^15^, ^16^ ^]^

**Figure 1:**
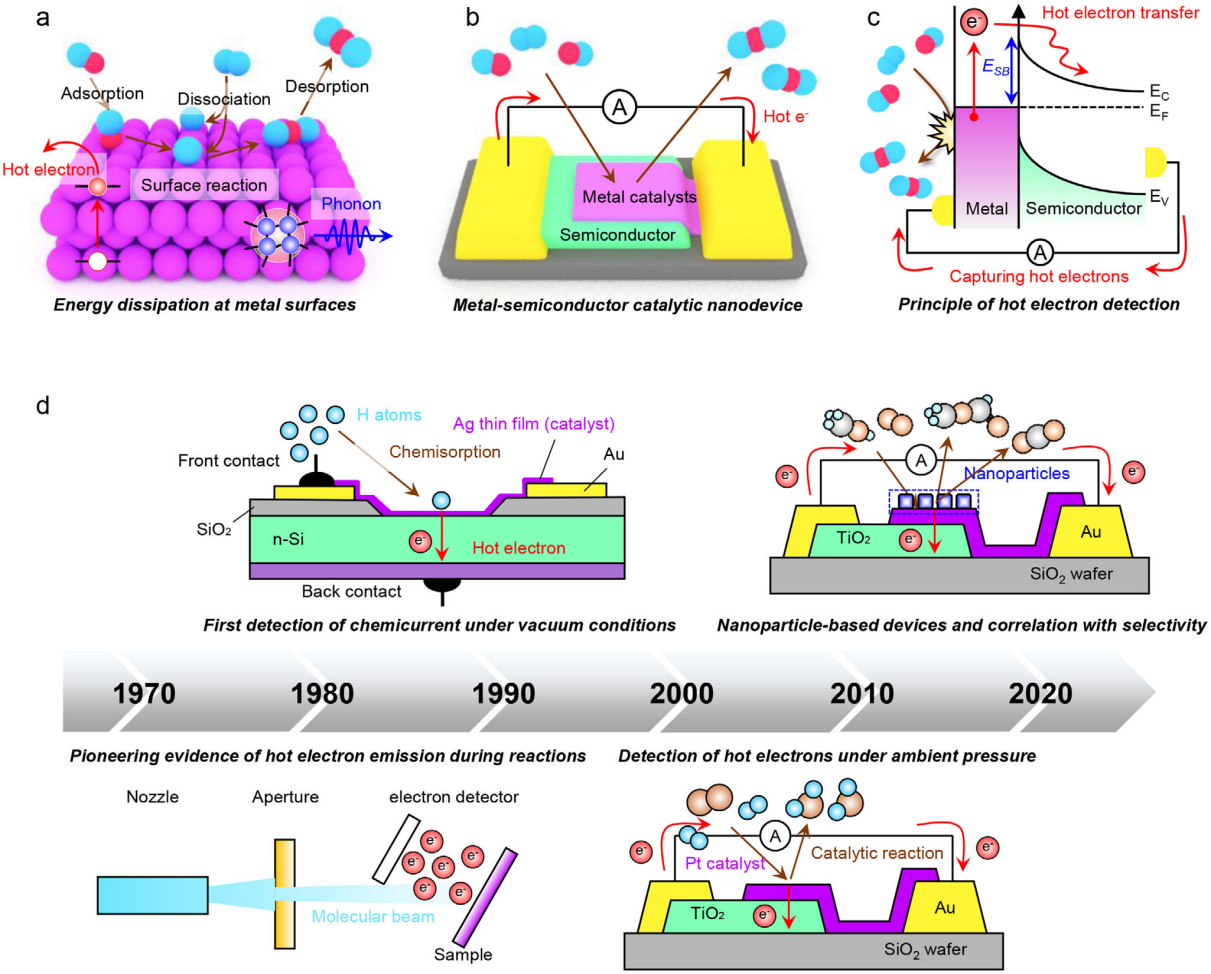
a) Energy dissipation pathways at metal surfaces during catalytic reactions. When a chemical reaction occurs on a metal surface, energy is dissipated through two primary pathways: non‐adiabatic electronic excitation, which generates hot electrons, and adiabatic dissipation, which leads to phonon excitation. b) Schematic of a metal–semiconductor catalytic nanodevice for hot electron detection. The device consists of a metal catalyst layer deposited on a semiconductor, enabling the direct measurement of hot electron flux through a chemicurrent, thereby providing real‐time insights into catalytic reaction dynamics. c) Principle of hot electron detection using a metal–semiconductor nanodiode. Hot electrons generated at the metal surface during catalytic reactions gain sufficient energy to overcome the Schottky barrier at the metal–semiconductor junction. This process allows their direct capture before thermalization, leading to measurable chemicurrents. d) Milestones in hot electron detection during surface reactions. From left to right: (1) early evidence of nonadiabatic electronic excitations using molecular beams and exoelectron detectors; (2) first detection of chemicurrent during hydrogen chemisorption on metal–semiconductor nanodiodes under vacuum; (3) transition to ambient‐pressure catalytic reactions, enabling correlation between hot electron signals and catalytic activity; and (4) integration of metal nanoparticles onto nanodiodes, facilitating hot electron detection in realistic catalyst geometries beyond planar thin films.

Recent studies have demonstrated that chemical reactions on metal surfaces can induce non‐adiabatic electronic excitations, resulting in the generation of hot electrons. Although such excitations occur infrequently and do not drive the reaction itself, these excitations, though limited in occurrence, arise as a byproduct of energy dissipation during exothermic surface reactions and provide insight into non‐adiabatic processes at catalytic interfaces.^[^ ^17^, ^18^, ^19^, ^20^, ^21^, ^22^ ^]^ As illustrated in **Figure** **1**a, when an exothermic reaction occurs at a metal surface, energy can be dissipated via two primary mechanisms: adiabatic and non‐adiabatic processes.^[^ ^23^, ^24^, ^25^, ^26^, ^27^, ^28^, ^29^, ^30^, ^31^, ^32^ ^]^ In the adiabatic process, energy is transferred to the lattice, exciting phonons and increasing the temperature of the catalyst.^[^ ^33^ ^]^ In contrast, the non‐adiabatic process involves energy transfer to conduction‐band electrons, generating hot electrons, which are excited well above the Fermi level with excess kinetic energy of ≈1–3 eV.^[^ ^11^, ^34^, ^35^ ^]^ These energetic charge carriers propagate within the metal and undergo inelastic scattering, primarily through electron–electron and electron–phonon interactions, ultimately relaxing back to the Fermi level.^[^ ^36^ ^]^

Hence, the transport of hot electrons is fundamentally constrained by their mean free path, which typically ranges from 1–10 nm, depending on the metal. To efficiently extract and detect these energetic carriers, ultrathin metal films are necessary, allowing hot electrons to reach an adjacent semiconductor before relaxation occurs.^[^ ^37^ ^]^ To enable real‐time detection of hot electrons, metal–semiconductor catalytic nanodiodes have been developed (Figure 1b). For the successful integration of catalytic systems with Schottky nanodiodes, several material requirements must be satisfied. First, the metallic film must serve as both the active catalytic site and the hot electron source, necessitating careful selection of metals appropriate for the targeted surface reaction (e.g., Pt for oxidation reactions or Pd for hydrogenation). Second, the catalytic metal should form a clean and stable Schottky contact with the underlying semiconductor, typically determined by the difference between the metal work function (Φ_m_) and the semiconductor electron affinity (χ), and modulated by Fermi level pinning effects.

The resulting Schottky barrier height (Φ_B_) should ideally fall within the range of 0.8–1.3 eV, which aligns with the kinetic energy window of reaction‐induced hot electrons. This range is sufficiently high to suppress undesired thermal electron flow across the junction, yet low enough to enable efficient detection of non‐thermal charge carriers excited by surface reactions. Furthermore, the metal–semiconductor junction must maintain rectifying behavior under realistic catalytic conditions, including elevated temperatures and gas‐phase reactants at ambient or higher pressures. These nanodevices consist of a thin catalytic metal layer deposited onto a semiconductor, forming a Schottky junction that selectively allows energetic charge carriers to be injected into the semiconductor if they possess sufficient energy to overcome the Schottky barrier (Figure 1c). The resulting chemicurrent provides a direct means to study charge transfer dynamics at catalytic interfaces, offering new insights into non‐adiabatic energy dissipation and reaction‐induced electronic excitations.

In this review, we highlight recent advances in hot electron transport and detection during catalytic reactions using metal–semiconductor catalytic nanodevices. We begin by revisiting the earliest discoveries of nonadiabatic electronic excitations on metal surfaces, such as exoelectron emission and surface chemiluminescence observed under ultrahigh vacuum. We then discuss the development of Schottky nanodiodes that enabled the first direct detection of hot electrons during hydrogen chemisorption under vacuum conditions. Building on this foundation, we describe how catalytic nanodiodes were adapted to probe real catalytic reactions—such as hydrogen and CO oxidation—at ambient pressures, allowing direct correlation between hot electron flow and catalytic turnover. Finally, we introduce recent studies that incorporate nanostructured catalysts, including supported metal nanoparticles, into nanodiode platforms to investigate complex, multi‐pathway reactions and selective energy dissipation. This chronological progression, outlined in Figure 1d, serves as a framework for the present review. Finally, we outline key challenges and future directions in utilizing hot electron transfer to gain deeper insights into energy dissipation phenomena during catalytic reactions and to develop advanced catalytic nanodevices.

## 2 Real‐Time Detection of Hot Electrons via Catalytic Nanodevices under High Vacuum Conditions

### 2.1 Pioneering Evidence of Nonadiabatic Electronic Excitations during Surface Reactions

The study of hot electron generation during chemical reactions at metal surfaces has its origins in the 1970s, when researchers began to recognize that energy dissipation during surface reactions could not be fully described by phonon excitation alone.^[^ ^38^ ^]^ This shift was motivated by the observation that certain exothermic reactions—such as the adsorption or dissociation of molecules on clean metal surfaces—produced signals that suggested the presence of excited charge carriers. Notably, several pioneering experiments conducted under ultrahigh vacuum conditions reported the emission of so‐called exoelectrons—low‐energy electrons emitted from metal surfaces during gas‐surface interactions, including systems such as Cs + O_2_ and Li + O_2_.^[^ ^39^, ^40^ ^]^ In parallel, researchers also detected surface chemiluminescence, where photon emission from metal surfaces was attributed to the radiative recombination of excited electrons and holes generated during chemical reactions.^[^ ^20^, ^29^, ^41^, ^42^ ^]^

These pioneering studies, while indirect in their detection methods, clearly demonstrated that surface reactions can excite electrons above the Fermi level, providing evidence for nonadiabatic electronic excitation during catalysis. They collectively established the concept that surface chemical processes can induce hot electron generation through nonadiabatic energy dissipation pathways—distinct from conventional adiabatic, lattice‐mediated mechanisms. These early findings laid the foundation for understanding how chemical reaction energy can be partially transferred into electronic degrees of freedom at catalytic interfaces.

### 2.2 Early Device‐Based Detection of Reaction‐Induced Hot Electron Transfer

**Figure 2:**
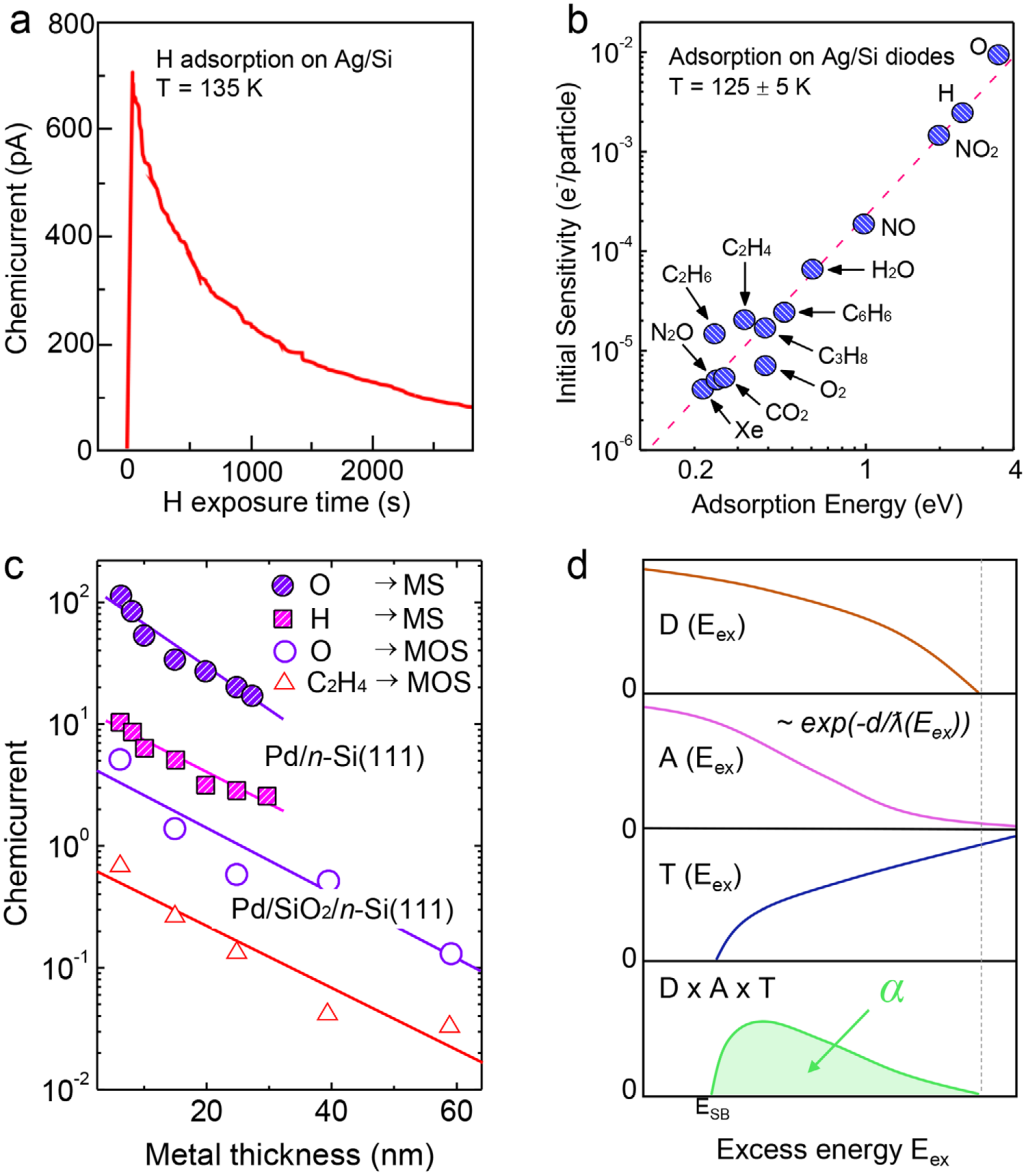
a) Time evolution of chemicurrent during H adsorption on Ag/Si. The gradual decrease in current suggests surface site saturation and reduced hot electron generation over time. Experiments were performed under ultrahigh vacuum conditions (base pressure ≈10^−8^ Torr) using a modulated atomic hydrogen beam generated by microwave plasma. The hydrogen flux was ≈7.5 × 10^11^ atoms s^−1^, and the surface temperature was maintained at 135 K. Reproduced with permission.^[^ ^43^ ^]^ Copyright 1999, American Physical Society. b) Relationship between initial chemicurrent sensitivity and adsorption energy for different gas species on Ag/Si diodes. Stronger interactions with the metal surface correlate with enhanced hot electron excitation. Measurements were conducted under ultrahigh vacuum (≈10^−10^ Torr) using modulated molecular beams of various gases (e.g., H, NO, NO_2_, H_2_O, Xe), with a beam flux of 2–4 × 10^13^ molecules cm^−2^ s^−1^. The substrate temperature was maintained at 125 ± 5 K. Reproduced with permission.^[^ ^17^ ^]^ Copyright 2001, American Association for the Advancement of Science. c) Chemicurrent as a function of metal thickness for Pd/n‐Si(111) and Pd/SiO_2_/n‐Si(111) Schottky nanodiodes. The exponential decay of chemicurrent with increasing metal thickness reflects the attenuation of hot electron transport, with differences between Pd/Si and Pd/SiO_2_/Si indicating the influence of interfacial electronic states. Experiments were carried out in a UHV chamber with a base pressure of 5 × 10^−8^ Torr. Reactant gases (e.g., H_2_, O_2_, CO_2_, C_2_H_4_, and Xe) were introduced as molecular beams with beam chamber pressures ranging from 15 to 80 Pa (≈0.1 to 0.6 Torr). The substrate temperature during measurements was 125 K. Reproduced with permission.^[^ ^46^ ^]^ Copyright 2004, American Physical Society. d) Schematic representation of the DAT (Distribution–Attenuation–Transmission) model describing chemicurrent generation. Hot electrons are generated during exothermic surface reactions with an energy distribution *D* (*E_ex_*), experience attenuation *A* (*E_ex_*) during transport across the metal layer, and contribute to the chemicurrent only if their energy exceeds the Schottky barrier height, with probability *T* (*E_ex_*). The product of these three energy‐dependent terms yields the overall chemicurrent contribution *α* (*E_ex_*), which peaks near *E_ex_* ≈*E_SB_* and decays at higher energies due to reduced generation probabilities. Reproduced with permission.^[^ ^37^ ^]^ Copyright 2002, Elsevier.

Building on this understanding, the development of Schottky nanodiodes by Nienhaus and co‐workers represented a pivotal advance. Their work enabled the direct electrical detection of reaction‐induced hot electrons via chemicurrent measurements, providing a practical and sensitive approach to monitor nonadiabatic electronic excitations at catalytic interfaces.^[^ ^37^, ^43^ ^]^ This work established the foundation for chemicurrent measurements, particularly under vacuum conditions, where fundamental interactions between adsorbates and metal surfaces can be studied without complications from mass transport effects. As shown in **Figure** **2**a, when hydrogen atoms adsorb onto the Ag surface, they induce localized charge transfer, leading to the excitation of conduction electrons via non‐adiabatic processes. These hot electrons propagate within the metal and, if they possess sufficient energy, traverse the metal film and overcome the Schottky barrier at the Ag/Si interface, injecting into the semiconductor and generating a measurable chemicurrent. This direct electronic response to surface reactions provides a means to study reaction‐induced energy dissipation at catalytic interfaces.

The time evolution of chemicurrent during hydrogen adsorption exhibits a characteristic decay over time. This decrease in chemicurrent is attributed to the gradual saturation of available adsorption sites, leading to a reduction in the hot electron generation rate. Such measurements provide insight into surface reaction kinetics and the dynamic nature of adsorption processes. Similar trends have been observed in studies examining chemicurrent responses under vacuum conditions, revealing a strong correlation between surface coverage and non‐adiabatic charge transfer efficiency.^[^ ^44^, ^45^ ^]^ Figure 2b further explores the relationship between chemicurrent response and adsorption energy for different gas species on Ag/Si nanodiodes.^[^ ^17^ ^]^ The observed correlation indicates that gas molecules with stronger metal‐surface interactions generate higher chemicurrent signals, suggesting a direct link between adsorption energetics and hot electron excitation efficiency. This finding highlights the potential of chemicurrent measurements as a quantitative tool for probing surface reaction energetics and provides insight into charge transfer dynamics at catalytic interfaces.

Moreover, Roldan et al. reported that chemicurrent measurements in Pd/n‐Si(111) Schottky and Pd/SiO_2_/n‐Si(111) metal‐oxide‐semiconductor (MOS) nanodevices show an exponential decay of the chemicurrent with increasing metal thickness (Figure 2c).^[^ ^46^, ^47^ ^]^ The trend is consistent with the attenuation of hot electron transport due to inelastic scattering processes within the metal film.^[^ ^14^ ^]^ Notably, while highly exothermic adsorption events such as hydrogen and oxygen dissociation generate detectable chemicurrent in both Schottky and MOS nanodiodes, low‐energy adsorption species (e.g., C_2_H_4_, CO_2_, and Xe) produce a measurable signal only in the MOS device. The difference arises from interfacial trap states at the metal‐oxide interface, which facilitate charge transfer through trapping and recombination of excited carriers. The MOS structure, by incorporating an ultrathin insulating layer between the metal and semiconductor, enables precise control of the energy barrier, allowing detection of lower‐energy chemical reactions. MOS nanodiodes provide an alternative approach to Schottky nanodiodes for detecting reaction‐induced charge transfer, particularly for species with low adsorption energies.

### 2.3 Quantifying Hot Electron Generation via Chemicurrent Yield

To understand and compare hot electron generation efficiencies across different catalytic systems, it is essential to establish a quantitative descriptor. The chemicurrent yield (α) serves this purpose by defining the number of reaction‐induced charge carriers detected per surface chemical event.^[^ ^5^, ^48^ ^]^ This quantity links catalytic turnover directly to nonadiabatic electronic excitation and can be experimentally determined by simultaneously measuring the chemicurrent and the molecular reaction rate. The chemicurrent yield is given by the expression:

(1) $$\alpha =\frac{I_{ch}}{e\cdot R}$$

where *I_ch_* is the current generated by hot electrons detected at the nanodiode, *e* is the elementary charge, and *R* is the rate of product formation in molecules per second. In catalytic systems where the turnover rate is further resolved into the product of active surface area, site density, and turnover frequency (TOF), the reaction rate can be expressed as *R* = *A*⋅*N*⋅TOF, yielding an alternative formulation of the chemicurrent yield:

(2) $$\alpha =\frac{I_{ch}}{e\cdot A\cdot N\cdot \mathrm{TOF}}$$

here, *A* is the geometric surface area of the catalyst, *N* is the density of catalytically active sites per unit area, and TOF is the number of product molecules formed per active site per second.^[^ ^15^, ^37^, ^49^ ^]^ This formulation enables a normalized comparison across systems with different catalyst morphologies, geometries, and site densities. Experimentally, the chemicurrent is measured under steady‐state or transient conditions using metal–semiconductor Schottky or MOS nanodiodes, while the reaction rate is typically determined via product quantification using gas chromatography or mass spectrometry. Reported values of *α* span several orders of magnitude—from ≈10^−6^ to 10^−1^ electrons per product molecule—depending on the metal identity, reaction energetics, device architecture, and interfacial properties.

To provide a mechanistic foundation for interpreting such variations, the Distribution–Attenuation–Transmission (DAT) model offers a physically grounded framework that describes the energy filtering and transport processes underlying chemicurrent generation.^[^ ^15^, ^37^, ^50^, ^51^ ^]^ According to this model, the overall yield of detected hot electrons is determined by the convolution of three energy‐dependent processes: 1) the generation of hot electrons with an energy distribution *D* (*E_ex_*), which reflects the excess energy available during exothermic surface reactions; 2) the attenuation of these electrons during transport through the metal layer, described by a decay function *A* (*E_ex_*) that accounts for inelastic scattering and mean free path limitations; and 3) the energy‐dependent transmission probability *T* (*E_ex_*) for hot electrons to overcome the Schottky barrier and be injected into the semiconductor. The total chemicurrent yield is thus expressed as:

(3) $$\alpha =\int_{0}^{\infty }D\left(E_{ex}\right)\;A\left(E_{ex}\right)\;T\left(E_{ex}\right)\;dE_{ex}$$

This formulation emphasizes that only electrons with energies exceeding the Schottky barrier height contribute to the chemicurrent signal. The overall efficiency is further modulated by physical parameters such as metal thickness, barrier height, and electronic scattering pathways within the metal. As illustrated in Figure 2d, the product of these three energy‐dependent functions yields a chemicurrent spectrum that typically peaks near the threshold energy required to overcome the barrier and decays at higher energies due to reduced electron population and scattering losses. The DAT model, therefore, provides a valuable framework for linking experimentally measured yields to microscopic energy dissipation and charge transport processes occurring at catalytic nanointerfaces.

These theoretical and methodological frameworks enable a systematic comparison of chemicurrent yields across diverse catalytic nanodevices. For hydrogen adsorption reactions, chemicurrent yields vary considerably depending on the metal identity. For instance, Ag/n‐Si nanodiodes yield ≈4.5 × 10^−3^ electrons per H atom, whereas Cu/n‐Si devices show a much lower value of ≈1.5 × 10^−4^ electrons per H atom under similar conditions—primarily due to the shorter mean free path of hot electrons in Cu and increased interfacial scattering.^[^ ^18^, ^43^ ^]^ Isotope substitution experiments further confirm the energetic sensitivity of hot electron generation: replacing H with D on Ag surfaces reduces the chemicurrent yield by a factor of ≈6,^[^ ^37^, ^43^ ^]^ consistent with lower excess energy release during D adsorption and hence fewer electrons exceeding the Schottky barrier.^[^ ^52^ ^]^

Structural engineering of the semiconductor support can also strongly influence chemicurrent behavior. In a catalytic nanodiode composed of a thin Pt film deposited on well‐ordered TiO_2_ nanotube arrays, a chemicurrent yield of ≈1.8 × 10^−4^ electrons per H_2_O molecule was achieved during hydrogen oxidation.^[^ ^53^, ^54^ ^]^ This yield is about one order of magnitude higher than that observed in a planar Pt/TiO_2_ nanodiode operated under identical conditions. The enhanced yield is attributed to more efficient hot electron transport through the vertically aligned TiO_2_ nanotubes, which reduce recombination losses and enable orthogonal charge collection. The reaction environment itself plays a decisive role in chemicurrent generation. In a recent comparative study of hydrogen peroxide decomposition over Pt/n‐Si nanodiodes, the chemicurrent yield increased from ≈10^−5^ electrons per O_2_ molecule in the gas phase to ≈10^−3^ in the aqueous phase—reflecting a nearly 100‐fold enhancement. This dramatic increase is attributed to Schottky barrier lowering by the electrolyte double layer and more efficient energy coupling at solid–liquid interfaces.^[^ ^16^ ^]^ Similar trends were observed in transient measurements reporting chemicurrent bursts of up to 0.12 electrons per O_2_ molecule in liquid‐phase reactions.^[^ ^55^ ^]^

Together, these comparisons reveal that chemicurrent yields span a wide dynamic range—from below 10^−6^ electrons per product molecule in weakly exothermic or poorly coupled systems, to ≈10^−1^ electron per product in optimized liquid‐phase devices. These variations reflect a complex interplay between reaction energetics, interfacial structure, and charge carrier transport properties. Quantitative benchmarking of chemicurrent yield across different catalytic environments thus provides critical mechanistic insight and informs the rational design of catalytic nanodiodes for operando studies.

## 3 Extending Chemicurrent Measurements to Ambient Conditions: Correlation with Catalytic Activity and Selectivity

### 3.1 Direct Correlation between Hot Electron Transfer and Catalytic Activity

**Figure 3:**
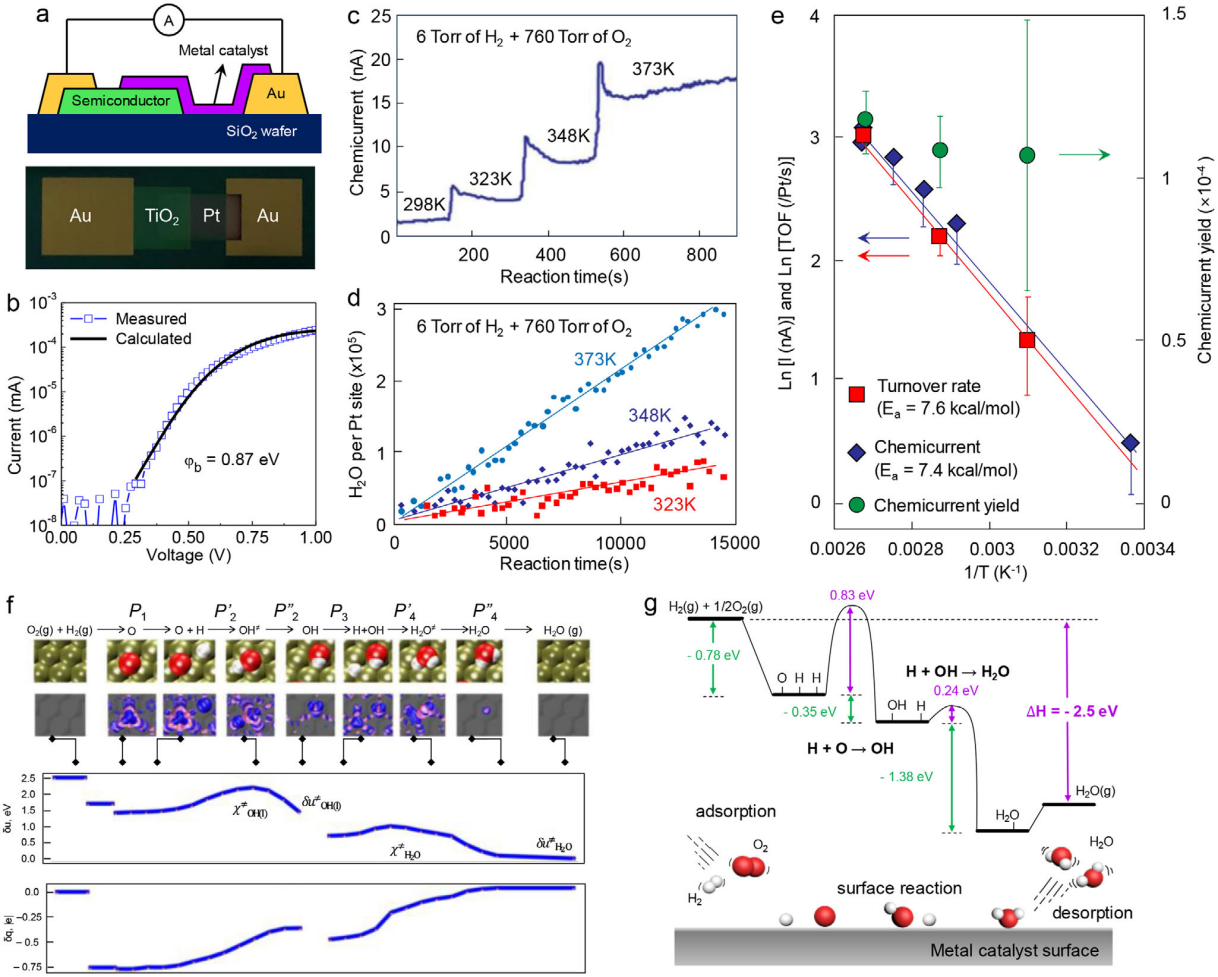
a) Schematic and optical image of the Pt/TiO_2_ Schottky nanodiode. The device consists of a Pt/TiO_2_ catalytic junction on a semiconductor substrate with Au contacts. b) Current–voltage characteristics of the Pt/TiO_2_ Schottky nanodiode. c) Time‐dependent chemicurrent measurements during hydrogen oxidation (6 Torr of H_2_ with O_2_ to 760 Torr) at different temperatures (298–373 K). d) Reaction kinetics of H_2_O formation on Pt sites at different temperatures. e) Arrhenius plot of chemicurrent and turnover frequency for hydrogen oxidation. Reproduced with permission.^[^ ^48^ ^]^ Copyright 2009, American Chemical Society. f) Reaction coordinate of the hydrogen oxidation process, showing the formation of key intermediates. Atomic configurations illustrate the sequential transformation from O_2_ and H_2_ gas‐phase reactants to surface‐bound intermediates, leading to the final desorption of H_2_O. The lower panels show the evolution of reaction energy and charge redistribution along the reaction coordinate, highlighting key transition states. Reproduced with permission.^[^ ^60^ ^]^ Copyright 2017, American Chemical Society. g) Energy diagram of the hydrogen oxidation reaction, illustrating the exothermic nature of the process and hot electron generation. Reproduced with permission.^[^ ^61^ ^]^ Copyright 2018, Springer Nature.

Early chemicurrent studies primarily focused on vacuum environments, where fundamental interactions between adsorbates and metal surfaces could be examined. However, the applicability of such studies to practical catalytic systems remained limited due to the substantial pressure gap between fundamental surface science experiments and real‐world catalytic conditions.^[^ ^56^ ^]^ Addressing this gap, Somorjai and co‐workers were the first to demonstrate that chemicurrent measurements could be successfully performed under ambient pressure reaction conditions, enabling direct correlation between catalytic turnover and hot electron generation.^[^ ^48^, ^57^, ^58^ ^]^ **Figure** **3**a shows the schematic structure of a Pt‐TiO_2_ catalytic nanodiode used for ambient pressure chemicurrent detection. Figure 3b presents the current–voltage (*I*–*V*) characteristics of the Pt/TiO_2_ nanodiode, exhibiting clear rectifying behavior typical of a Schottky junction. To quantify the barrier properties, the forward‐biased *I*–*V* curve was fitted using the thermionic emission model: $I=I_{S}\;\left[\exp(\frac{e(V-IR_{S})}{\eta k_{B}T})-1\right]$, where *I_S_* is the saturation current ($I_{S}=AA^{*}T^{2}\exp\left(-\frac{e\varphi_{b}}{k_{B}T}\right)$), η is the ideality factor, and φ_*b*_ is the Schottky barrier height. From this fitting, the Schottky barrier height was determined to be ≈0.82 eV, confirming the formation of a stable metal–semiconductor junction suitable for hot electron detection. While the pressure and temperature conditions used in these chemicurrent studies (≈1 bar, 100–150 °C) remain modest compared to industrial catalytic processes (10–400 bar, 400–500 °C), they represent a substantial advance from the ultra‐high vacuum environments (≈10^−6^ to 10^−9^ Torr) employed in early chemicurrent measurements. This progression has enabled a more realistic simulation of catalytic reaction environments within operando nanodevice platforms.

The correlation between chemicurrent and catalytic turnover can provide direct insights into reaction‐induced electronic excitations. The temperature dependence of chemicurrent during ambient pressure hydrogen oxidation reveals an increase in signal intensity as the reaction temperature rises (Figure 3c), demonstrating that the stepwise nature of the response suggests the increasing chemicurrent response at elevated temperatures reflects the higher reaction rates of hydrogen oxidation and the enhanced probability of electronic excitation events. Figure 3d displays the catalytic activity of the Pt surface during hydrogen oxidation, expressed as the number of water molecules produced per Pt site over time, revealing a clear temperature dependence. To quantify the relationship between reaction energetics and hot electron excitation, Figure 3e shows an Arrhenius analysis, and the activation energy extracted from chemicurrent measurements (7.4 kcal mol^−1^) closely matches that obtained from catalytic turnover rates (7.6 kcal mol^−1^), indicating that hot electron excitation is directly linked to the rate‐determining step of hydrogen oxidation. Moreover, the chemicurrent yield, defined as the fraction of catalytic events producing hot electrons, remains relatively constant across the investigated temperature range. A similar trend has been observed in other ambient pressure reaction studies, such as CO oxidation on Pt/TiO_2_ nanodevices,^[^ ^57^, ^59^ ^]^ suggesting that chemicurrent may serve as a qualitative indicator of surface reaction energetics and non‐adiabatic excitations.

Computational investigations have provided significant insights into the electronic excitations associated with hydrogen oxidation on catalytic metal surfaces, and theoretical studies by Maximoff et al. employed ab initio molecular dynamics and density functional theory (DFT) calculations to examine the role of charge transfer in reaction‐induced hot electron generation.^[^ ^60^ ^]^ As shown in Figure 3f, the reaction pathway of hydrogen oxidation on Pt surfaces reveals distinct charge fluctuations at key intermediate states, and a key mechanistic feature is the electronic excitation occurring during the dissociation of molecular oxygen and the subsequent formation of hydroxyl (OH) and water (H_2_O) intermediates.^[^ ^61^ ^]^ Specifically, Figure 3f visualizes real‐space charge redistribution during the reaction process. The contour maps highlight the accumulation and depletion of electronic density at the Pt(111) interface as H^−^ and O^−^ react to form OH^−^ and H_2_O. Simultaneously, the reaction energy profile shows exothermic steps coinciding with sharp charge transfer events. During these transitions, a portion of the localized charge on the adsorbates is released back into the metal, suggesting non‐adiabatic electron injection into conduction states. This analysis highlights the fact that chemicurrent generation stems from non‐adiabatic electron transfer at high‐energy intermediates, rather than from bulk heating effects. The dynamic discharge of electrons from transiently charged surface species‐especially during the formation of OH and H_2_O‐supports the concept of a surface electron pump, wherein redox‐driven electron flow across the metal interface gives rise to hot electron emission.

Charge redistribution during the reaction pathway was further analyzed through projected density of states calculations, revealing that specific reaction steps induce significant shifts in the local electronic structure of the metal surface. Figure 3g further conceptualizes reaction pathways of H_2_ oxidation on the Pt surface, and among the elementary reaction steps, the reaction of H and OH to form H_2_O exhibits the highest exothermic energy release. The substantial energy released in this reaction has a higher probability of generating hot electrons that can be injected into the semiconductor substrate, leading to the observed chemicurrent signals in Figure 3c. This reaction step (H + OH → H_2_O), which releases ≈−1.38 eV, provides enough energy to overcome the Schottky barrier at the metal–semiconductor interface, making it the most probable origin of hot electron generation. Furthermore, the formation of volatile H_2_O allows the Pt surface to regenerate its active sites, enabling steady‐state catalysis and continuous chemicurrent under ambient conditions. This interpretation is supported by experimental evidence showing that chemicurrent signals track closely with turnover frequency, and isotope‐substitution studies (H_2_ vs D_2_) confirm that the rate‐determining step is tightly coupled to the energetics of the H + OH association.^[^ ^61^ ^]^ Overall, ambient pressure chemicurrent detection enables the direct investigation of energy dissipation pathways in working catalysts. The ability to correlate hot electron transport with catalytic activity in real time represents a significant step forward in understanding the role of non‐adiabatic charge transfer in heterogeneous catalysis. Ongoing efforts continue to refine these techniques, expanding their applicability to a broader range of catalytic systems and reaction environments.

### 3.2 Selectivity‐Dependent Efficiency of Hot Electron Generation

**Figure 4:**
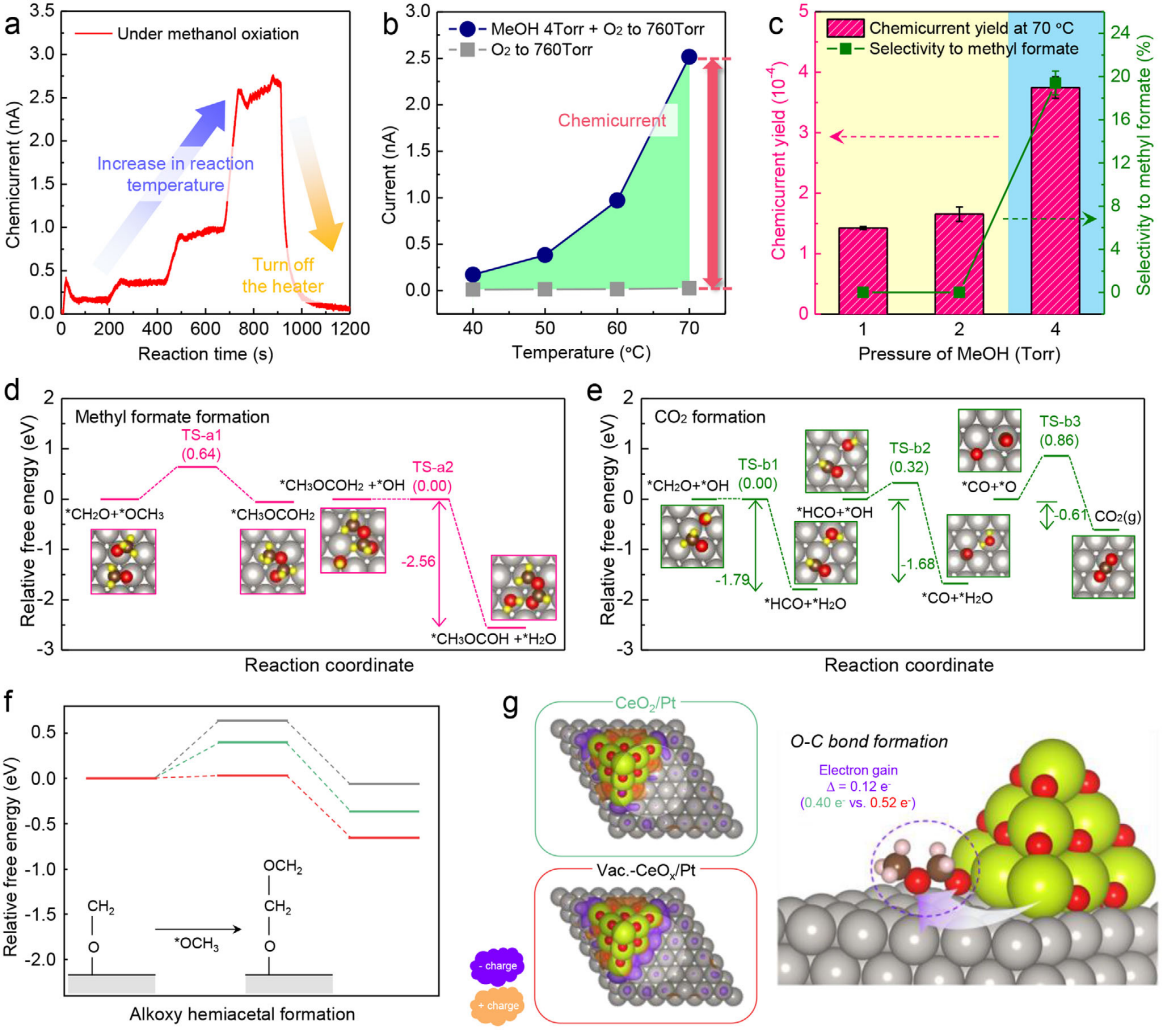
a) Time‐dependent chemicurrent measurement during methanol oxidation (4 Torr of MeOH with O_2_ to 760 Torr) on Pt/n‐TiO_2_ Schottky nanodiodes. The increase in chemicurrent with rising reaction temperature indicates enhanced hot electron generation due to higher catalytic activity. b) Steady‐state chemicurrent as a function of temperature under methanol oxidation and pure oxygen conditions. The net chemicurrent is obtained by subtracting the thermoelectric contribution measured in pure O_2_, confirming that the signal originates from reaction‐induced hot electron transport. c) Chemicurrent yield and methyl formate selectivity as a function of methanol pressure. The correlation between chemicurrent yield and selectivity suggests that hot electron generation is linked to the reaction pathway favoring partial oxidation. d,e) Reaction energy profiles for methyl formate and CO_2_ formation pathways on Pt/TiO_2_. The formation of methyl formate exhibits the highest exothermic energy release among the reaction steps, suggesting that partial oxidation contributes more significantly to hot electron generation than complete oxidation to CO_2_. Reproduced with permission.^[^ ^19^ ^]^ Copyright 2019, American Chemical Society. f) Free energy profiles for the key C─O coupling step (^*^CH_3_O + ^*^CH_2_O → ^*^CH_3_OCOH_2_) in the methyl formate formation pathway calculated on Pt(111), CeO_2_/Pt(111), and Vac.‐CeO_x_/Pt(111) surfaces. g) Charge density difference maps showing electron redistribution at the oxide–metal interface. Reproduced with permission.^[^ ^64^ ^]^ Copyright 2018, Springer Nature.

While previous studies have primarily focused on the relationship between catalytic activity and hot electron excitation, recent research has demonstrated that reaction selectivity can also influence non‐adiabatic energy dissipation. Park and co‐workers were the first to experimentally establish the correlation between catalytic selectivity and hot electron generation using Pt/TiO_2_ catalytic nanodevices.^[^ ^19^, ^36^ ^]^ Their pioneering work demonstrated that selective oxidation reactions on metal surfaces could be directly linked to non‐adiabatic charge transfer events, extending the scope of chemicurrent studies beyond catalytic activity to include reaction selectivity. The time‐dependent chemicurrent response during methanol oxidation at ambient pressure, as shown in **Figure** **4**a, exhibits a steady increase in current as the reaction temperature rises. Figure 4b shows the steady‐state chemicurrent response as a function of reaction temperature, demonstrating that the presence of methanol under oxidative conditions induces a significantly higher chemicurrent than pure oxygen alone.

The relationship between chemicurrent yield and catalytic selectivity is further examined in Figure 4c. A strong correlation is observed between selectivity toward methyl formate and the measured chemicurrent yield. Prior studies have shown that methanol oxidation follows two primary pathways: full oxidation to CO_2_ and partial oxidation to methyl formate.^[^ ^62^, ^63^ ^]^ Experimental results indicate that the methyl formate pathway leads to a significantly higher chemicurrent yield, implying that the non‐adiabatic energy dissipation process is more pronounced in this reaction channel. These findings are supported by theoretical calculations, as shown in Figure 4d,e, where DFT calculations reveal the energy profiles of the two reaction pathways on a Pt(111) surface. The formation of methyl formate involves a highly exothermic reaction step, which aligns well with experimental chemicurrent measurements, further supporting the hypothesis that the extent of electronic excitation is intrinsically linked to the exothermicity of individual reaction steps. Specifically, the final step in the methyl formate pathway‐^*^CH_3_OCOH_2_ + ^*^OH → CH_3_OCOH(g) + ^*^H_2_O‐was calculated to release 2.56 eV of energy, while the most exothermic step in the CO_2_ formation pathway (^*^CH_2_O + ^*^OH → ^*^HCO + H_2_O) releases only 1.79 eV. Both values exceed the Schottky barrier height (≈0.87 eV) at the Pt/TiO_2_ junction, enabling hot electron detection; however, the greater energy release in the methyl formate path rationalizes the enhanced chemicurrent observed in experiments. These results suggest that hot electron generation is not only a function of total reaction energetics but is highly sensitive to the energy release of individual steps, particularly those involving bond formation and product desorption. Overall, the observed correlation between selectivity and chemicurrent yield highlights the critical role of non‐adiabatic charge transfer in governing catalytic selectivity and suggests that non‐adiabatic excitations may provide additional insight into surface‐level energy dissipation phenomena, complementing existing tools for studying interfacial charge dynamics during catalytic reactions.

While the preceding discussion focused on detecting reaction‐induced charge transfer through chemicurrent measurements, a deeper understanding of how electron transfer affects catalytic selectivity requires theoretical insight into interfacial reaction energetics. In a recent report, nanowire‐integrated nanodiodes incorporating CeO_x_–Pt interfaces exhibited both enhanced methyl formate selectivity and increased chemicurrent yields, suggesting a potential correlation between interfacial electronic structure and preferred reaction pathways.^[^ ^64^ ^]^ To explore this correlation, DFT calculations were conducted to compare the activation barriers for the alkoxy hemiacetal formation step (^*^CH_3_O + ^*^CH_2_O → ^*^CH_3_OCOH_2_) on Pt(111), CeO_2_/Pt(111), and vacancy‐rich CeO_x_/Pt(111) surfaces. As shown in Figure 4f, the barrier was significantly lower for the Vac.‐CeO_x_/Pt system (0.032 eV) compared to CeO_2_/Pt (0.398 eV) and Pt (0.634 eV), aligning with the experimentally observed trend in selectivity. This result highlights the critical influence of interfacial environments on reaction energetics and provides a plausible explanation for the enhanced performance of vacancy‐rich oxide systems.

Charge density difference analysis (Figure 4g) further revealed that the oxygen‐vacancy‐rich interface facilitates electron accumulation near the reacting intermediates. Bader charge analysis indicated a net electron gain of ≈0.12 e^−^ relative to the stoichiometric CeO_2_/Pt interface, suggesting that excess interfacial electrons can stabilize the transition state and lower the activation barrier. These findings support the notion that interfacial electron redistribution, particularly at engineered metal–oxide junctions, plays an important role in modulating selectivity during exothermic oxidation reactions.

A similar conclusion was reached in a separate study involving Pt nanoparticles supported on TiO_2_ of different morphologies, where shape‐controlled anatase TiO_2_ nanocrystals with abundant (001) facets promoted enhanced methyl formate formation during methanol oxidation.^[^ ^62^ ^]^ DFT calculations revealed that the Pt/TiO_2_ (001) interface exhibited the strongest charge transfer to the HCOOCH_3_ intermediate, correlating with higher binding energies and lower formation energies compared to Pt(111) or Pt/TiO_2_ (101) surfaces. These results collectively underscore the critical role of oxide–metal interfaces and facet‐dependent electronic interactions in steering reaction pathways and stabilizing desirable products.

Together, these studies suggest that non‐adiabatic charge transfer processes are not merely byproducts of catalytic reactions but may actively participate in shaping reaction selectivity. The ability to correlate interfacial electronic properties with bond‐forming reaction steps offers a useful framework for designing catalysts that combine efficient hot electron generation with improved molecular‐level control over chemical transformations.

### 3.3 Addressing Thermoelectric Contributions under Ambient‐Pressure Conditions

Chemicurrent detection using catalytic nanodiodes has emerged as a powerful strategy for probing nonadiabatic electronic excitations at catalytic interfaces. In particular, these devices allow for the direct electrical measurement of hot electron flow generated during exothermic surface reactions. While early studies were primarily conducted under ultrahigh vacuum and isothermal conditions, where extrinsic thermal effects were negligible, recent efforts to extend this methodology to ambient‐pressure environments have introduced new complications. As the operating temperature and pressure increase to approach realistic catalytic conditions, the potential influence of heat‐induced artifacts becomes nontrivial and must be carefully considered when interpreting chemicurrent data.^[^ ^45^ ^]^

**Figure 5:**
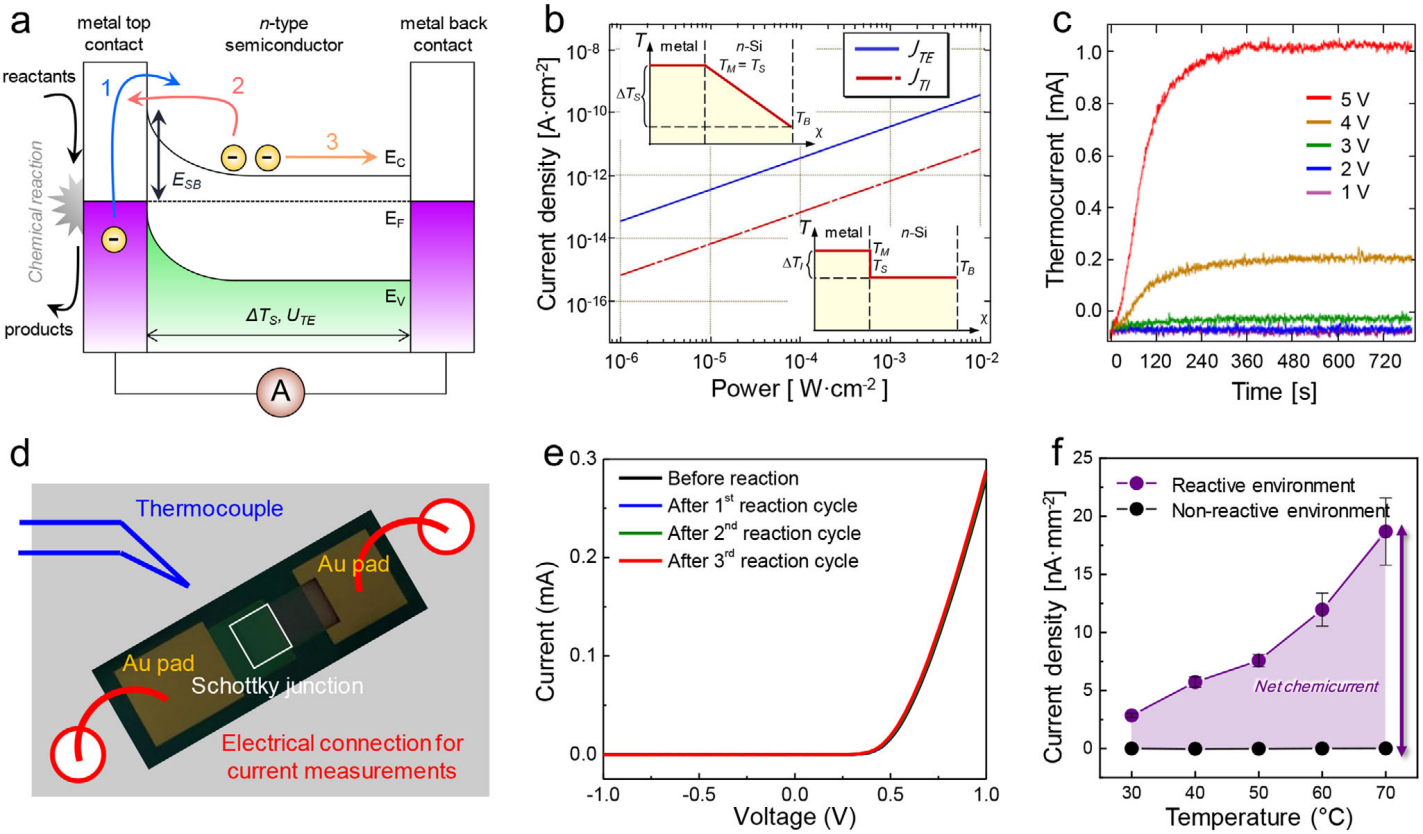
a) Schematic illustration of the three major charge transport mechanisms across a metal–semiconductor Schottky junction under catalytic conditions: (1) thermionic emission over the Schottky barrier, (2) hot electron chemicurrent generated by nonadiabatic energy dissipation, and (3) thermoelectric current induced by temperature gradients across the semiconductor. Reproduced with permission.^[^ ^45^ ^]^ Copyright 2016, The Royal Society of Chemistry. b) Theoretical estimation of thermoelectric current and thermionic emission current as a function of heating power, indicating that thermoelectric current can dominate under typical catalytic conditions. c) Experimental measurement of thermocurrent in a Pt/SiO_2_/*n*‐Si nanodiode under various heating voltages in the absence of any chemical reaction. The current follows the applied thermal profile, confirming its thermoelectric origin. Reproduced with permission.^[^ ^65^ ^]^ Copyright 2013, American Institute of Physics. d) Schematic diagram of a nanodiode measurement configuration designed to suppress thermoelectric artifacts. The device is fabricated on a SiO_2_ wafer to ensure electrical insulation from the heater, and the thermocouple used for temperature monitoring is positioned near—but not in contact with—the nanodiode. Current is measured exclusively across the Schottky junction via Au electrodes. Reproduced with permission.^[^ ^66^ ^]^ Copyright 2014, American Institute of Physics. e) *I–V* characteristics of a Pt/TiO_2_ nanodiode recorded before and after three repeated hydrogen oxidation cycles (15 Torr of H_2_ with O_2_ to 760 Torr), showing negligible change in diode behavior and confirming the structural and electronic stability of the junction. f) Comparison of current density as a function of temperature under reactive (15 Torr of H_2_ with O_2_ to 760 Torr) and non‐reactive (760 Torr of O_2_) environments. The observed increase in current under reactive conditions and the minimal signal in non‐reactive environments confirm that the dominant contribution arises from reaction‐induced hot electron excitation, not from thermoelectric background effects.

Among these artifacts, thermoelectric current represents a particularly significant source of potential error. In metal–semiconductor nanodiode structures, the establishment of a temperature gradient across the device—either laterally or vertically—can drive charge carriers via the Seebeck effect. This phenomenon results in the development of a thermoelectric voltage across the junction, even in the absence of any catalytic reaction. The resulting current can mimic or even exceed the magnitude of the reaction‐induced chemicurrent, thereby obscuring the true signal. Additionally, temperature‐dependent changes in Schottky barrier height or leakage current can further complicate current interpretation, especially under dynamic heating conditions. These concerns are not merely theoretical. As schematically illustrated in **Figure** **5**a,b, Nedrygailov and co‐workers provided a comparative analysis of thermionic and thermoelectric current contributions in typical nanodiode systems, demonstrating that thermoelectric current can dominate over thermionic emission by up to two orders of magnitude under catalytic operating conditions.^[^ ^65^ ^]^ Furthermore, as shown in Figure 5c, Meyburg et al. experimentally measured thermoelectric currents in Pt/SiO_2_/n‐Si devices in the absence of any reactants, revealing that externally applied heating alone is sufficient to generate substantial measurable current—purely due to thermal gradients across the substrate.^[^ ^66^ ^]^

A particularly relevant example is the work of Creighton and Coltrin, who investigated reaction‐induced current in Pt/GaN nanodiodes during CO oxidation at temperatures of 270–300 °C.^[^ ^67^ ^]^ Their findings showed that the observed current could be quantitatively explained by thermoelectric voltage generated from lateral temperature gradients, rather than hot electron excitation. These results highlight the inherent difficulty of chemicurrent measurements at high temperatures, where thermal effects are often unavoidable. In this context, selecting reaction systems that proceed efficiently at lower temperatures—such as H_2_ oxidation and methanol oxidation—provides a practical pathway to minimizing thermoelectric contributions. These reactions typically occur between 40 and 70 °C, a temperature range where device heating is modest and lateral gradients are greatly reduced. Such conditions allow for more confident attribution of measured current to reaction‐induced hot electron generation.

In addition to temperature control, system‐level experimental design plays a key role in suppressing thermal artifacts. As illustrated in Figure 5d, catalytic nanodiodes can be fabricated on thermally conductive but electrically insulating SiO_2_ wafers, which are placed on resistive heaters. This configuration allows for efficient thermal contact while maintaining electrical isolation between the heater and the nanodiode. Importantly, the heater voltage is not directly applied to the device, thereby eliminating the possibility that heating power induces spurious electrical signals. Moreover, thermocouples are positioned close to the diode—yet without making contact—to enable temperature monitoring without affecting current measurements.

Device stability under reaction conditions is another essential factor in validating chemicurrent data. As shown in Figure 5e and I–V curves measured before and after repeated reaction cycles reveal consistent rectifying behavior and unchanged Schottky barrier properties. These results confirm that the device maintains its electrical characteristics during catalysis, further excluding the possibility that current changes are due to junction degradation or temperature‐induced barrier shifts. Lastly, to isolate reaction‐induced current from thermal background contributions, chemicurrent responses are often compared under reactive and non‐reactive gas environments. As demonstrated in Figure 5f, significantly higher current is observed under reactive conditions compared to non‐reactive environments (e.g., He or O_2_), despite identical thermal profiles. This contrast enables the definition of a “net chemicurrent” as the differential current signal, reinforcing the conclusion that the measured charge flow is directly linked to the occurrence of chemical reactions at the catalyst surface.

Together, these strategies—including device engineering, reaction temperature selection, and control experiments—demonstrate how thermoelectric artifacts can be effectively minimized in ambient‐pressure chemicurrent measurements. The development and implementation of such approaches have been instrumental in reinforcing the credibility of hot electron detection and will continue to shape the design of future experiments aimed at quantifying energy dissipation pathways in heterogeneous catalysis.

## 4 Toward Real Catalysts: Detecting Hot Electrons in Nanoparticle‐Based Catalytic Systems

**Figure 6:**
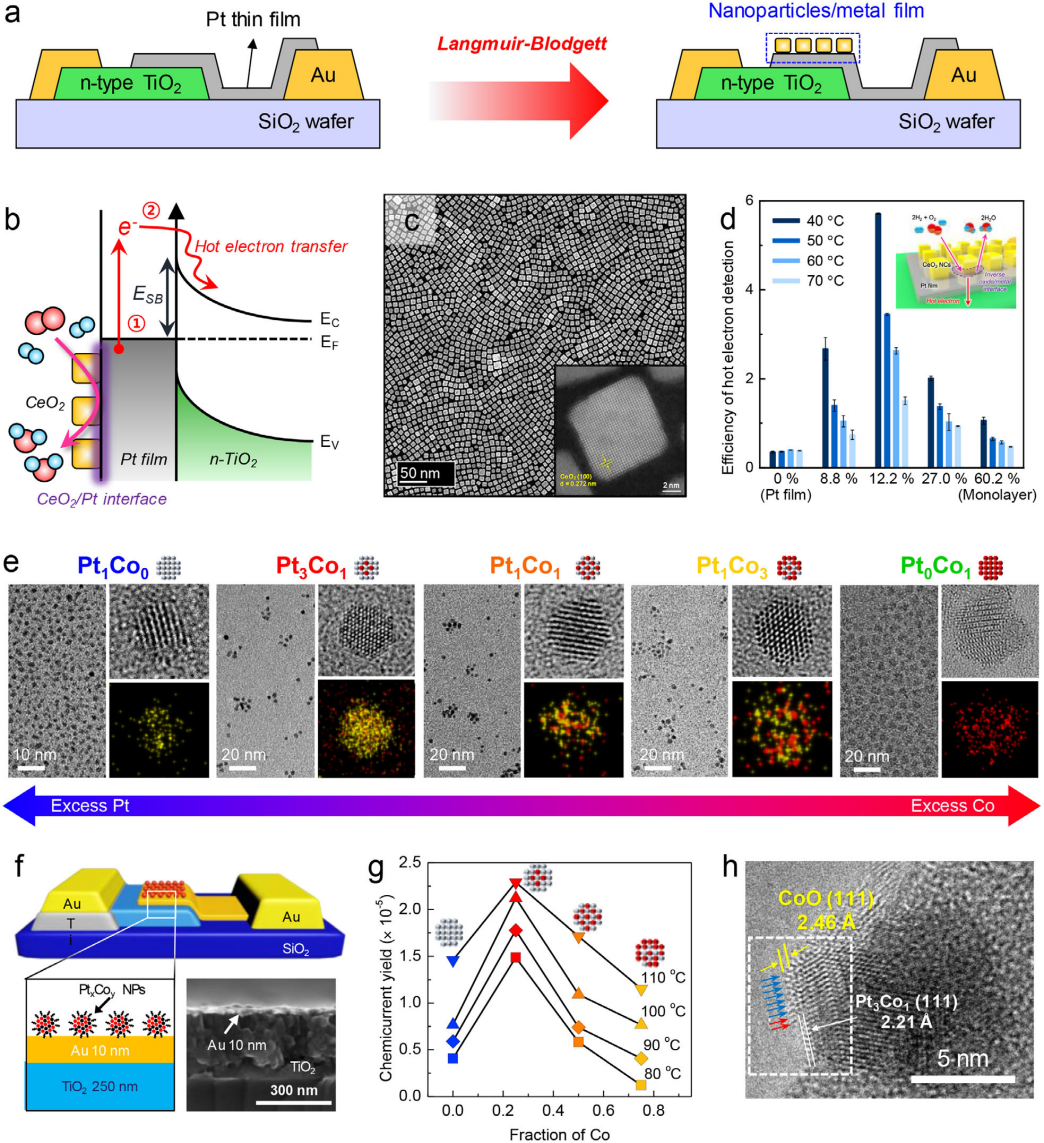
a) Schematic illustration of the fabrication process for CeO_2_/Pt/TiO_2_ Schottky nanodiodes using the Langmuir–Blodgett method. A monolayer of CeO_2_ nanoparticles is deposited onto a Pt thin film, forming a well‐defined oxide–metal interface. b) Energy band diagram of the CeO_2_/Pt/TiO_2_ nanodiode illustrating hot electron transfer across the Schottky barrier. Exothermic catalytic reactions at the CeO_2_/Pt interface generate hot electrons, which are injected into the n‐type TiO_2_ substrate. c) TEM image of the CeO_2_ nanoparticles deposited on the Pt film, showing uniform nanoparticle distribution and crystalline structure. The inset highlights a high‐resolution image of an individual CeO_2_ nanoparticle. d) Efficiency of hot electron injection as a function of temperature and CeO_2_ coverage. Chemicurrent yield was obtained during hydrogen oxidation (15 Torr of H_2_ with O_2_ to 760 Torr) conducted. The increasing fraction of CeO_2_ coverage enhances hot electron generation, with the highest injection efficiency observed at moderate coverage levels. Reproduced with permission.^[^ ^69^ ^]^ Copyright 2024, American Chemical Society. e) High‐angle annular dark‐field scanning transmission electron microscopy images and EDS elemental mapping of Pt_x_Co_y_ nanoparticles with varying Pt/Co ratios. f) Schematic illustration of the Pt–Co nanoparticle integration onto Au/TiO_2_ Schottky nanodiodes. g) Chemicurrent yield as a function of Pt–Co composition, showing that an optimal Pt/Co ratio maximizes hot electron generation. Chemicurrent yield was obtained during hydrogen oxidation (15 Torr of H_2_ with O_2_ to 760 Torr) conducted. h) In situ TEM observation of structural evolution in Pt_3_Co_1_ nanoparticles under 0.5 mbar O_2_ gas conditions. Reproduced with permission.^[^ ^71^ ^]^ Copyright 2018, Springer Nature.

The studies discussed thus far have primarily focused on thin‐film‐based catalytic nanodiodes for detecting hot electrons. However, real catalysts predominantly consist of nanoscale particles rather than continuous thin films. To bridge this material gap, Lee and co‐workers introduced a novel approach utilizing nanoparticles integrated into metal–semiconductor Schottky nanodiodes,^[^ ^49^, ^68^, ^69^ ^]^ and these studies demonstrated that hot electron detection could be effectively studied using nanostructured catalysts. As illustrated in **Figure** **6**a, the experimental setup involves depositing nanoparticles onto the Schottky junction of the conventional thin‐film‐based nanodiodes through the Langmuir–Blodgett method. As shown in Figure 6b, unlike conventional thin‐film‐based Schottky nanodiodes, the incorporation of oxide nanocubes onto the Pt film creates well‐defined oxide–metal interfaces, which significantly influence charge transfer dynamics. High‐resolution transmission electron microscopy (HR‐TEM) reveals the uniform dispersion of CeO_2_ nanocubes on the Pt/TiO_2_ nanodiode.

Figure 6d presents the efficiency of hot electron excitation as a function of deposition coverages. A non‐monotonic trend in chemicurrent yield is observed with increasing CeO_2_ coverage, indicating that the introduction of oxide nanocubes enhances hot electron generation up to an optimal coverage. Beyond this threshold, excessive CeO_2_ deposition results in a decline in efficiency, likely due to reduced access of reactants to the oxide‐metal interface. These findings suggest that the oxide‐metal interface plays a critical role in facilitating electron transfer and that an optimal balance between metal and oxide coverage is necessary for maximizing hot electron yield. Notably, the role of oxide‐metal interfaces in enhancing hot electron generation has been observed in other catalytic systems as well. Studies on Co_3_O_4_ nanocubes deposited onto Pt/TiO_2_ nanodiodes revealed a similar enhancement in chemicurrent yield, further supporting the idea that oxide‐metal interfaces serve as active sites for non‐adiabatic charge transfer.^[^ ^68^, ^70^ ^]^ Additionally, modifying the TiO_2_ support from a planar thin film to a mesoporous structure has been shown to accelerate hot electron transport, as the increased interfacial area between Pt and TiO_2_ facilitates more efficient charge carrier movement.^[^ ^53^ ^]^ The mesoporous TiO_2_ structure enhances the accessibility of reactants to the Pt/TiO_2_ interface and improves electron mobility, leading to an increase in chemicurrent efficiency.^[^ ^54^ ^]^ These observations highlight the broader applicability of oxide–metal interfaces in controlling electronic excitations and catalytic performance.

Moreover, Lee et al. systematically investigated the influence of bimetallic Pt‐Co nanoparticles on hot electron excitation during catalytic hydrogen oxidation using Au/TiO_2_ Schottky nanodiodes.^[^ ^71^ ^]^ Unlike previous studies utilizing monometallic nanoparticles, their work demonstrated that modifying the elemental composition of bimetallic catalysts can significantly alter chemicurrent efficiency by tuning both the electronic and catalytic properties of the metal surface. Transmission electron microscopy (TEM) and energy‐dispersive X‐ray spectroscopy (EDS) mapping reveal the structural and compositional evolution of Pt_1_Co, Pt_3_Co_1_, Pt_1_Co_1_, Pt_1_Co_3_, and Pt_0_Co_1_ nanoparticles (Figure 6e). A compositional gradient from Pt‐rich to Co‐rich nanoparticles was established, allowing for a systematic study of bimetallic effects on catalytic charge transfer. Figure 6f presents the experimental setup, where bimetallic Pt‐Co nanoparticles were deposited onto Au/TiO_2_ nanodiodes using the Langmuir–Blodgett method, ensuring precise control over nanoparticle monolayer formation.

The influence of Pt‐Co composition on chemicurrent yield is quantitatively examined in Figure 6g, notably, the Pt_3_Co_1_ composition exhibits the highest chemicurrent efficiency, indicating that an optimal balance of Pt and Co enhances hot electron generation. X‐ray photoelectron spectroscopy experiments revealed that during H_2_ oxidation, Co undergoes partial oxidation to form CoO. Interestingly, as the Co fraction increases, the amount of CoO formed also increases after reaction, which reduces the interfacial area between CoO and Pt. This decrease in interfacial area weakens the synergistic effect between the metal and oxide, thereby diminishing the catalytic activity and hot electron excitation efficiency at high Co loadings. In situ TEM image in Figure 6h confirms the structural transformation of Pt‐Co nanoparticles under reaction conditions, showing the formation of a CoO/Pt interface. This interface serves as an active site for catalytic reactions and non‐adiabatic charge transfer, contributing to the observed enhancement in chemicurrent yield at the optimal Pt_3_Co_1_ composition. However, at higher Co fractions, excessive oxidation leads to a loss of active CoO/Pt interfacial regions, which negatively impacts hydrogen oxidation kinetics. Hence, this study proves the importance of bimetallic nanoparticle composition in tailoring catalytic charge transfer processes. Overall, these works highlight the significance of oxide‐metal interfaces in catalytic hot electron generation and provide a framework for extending chemicurrent studies beyond planar thin‐film catalysts.

Although our nanocatalyst‐integrated nanodiode structures remain planar, this design reflects a necessary compromise to preserve electrical connectivity between the catalyst and external electrodes. In 3D catalysts (i.e., nanocatalysts incorporated in porous supports), metal nanoparticles are often electrically isolated, preventing the collection of hot electrons as current. By depositing nanocatalysts such as Pt, CeO_2_, or bimetallic alloys onto thin‐film Schottky devices, we maintain this critical electrical pathway while approximating the catalytic surface heterogeneity of real systems. By incorporating nanostructured catalysts into Schottky nanodiodes, this approach paves the way for more realistic investigations of charge transfer processes in heterogeneous catalysis.

## 5 From Detection to Control: Photon‐ and Bias‐Induced Modulation of Catalytic Reactions

### 5.1 Photon‐Induced Charge Transfer for Catalytic Modulation: A Reverse of Reaction‐Driven Hot Carrier Generation

While the preceding sections have examined the generation and detection of hot carriers originating from exothermic chemical reactions at metal–semiconductor junctions, the inverse approach—modulating catalytic activity through photoexcited hot carriers—offers a complementary perspective grounded in recent developments in light‐driven nanocatalysis. In particular, this section discusses the detailed mechanisms by which hot electrons and hot holes generated in plasmonic metal–semiconductor Schottky junctions can influence surface reactions, drawing on both experimental observations and theoretical models.

**Figure 7:**
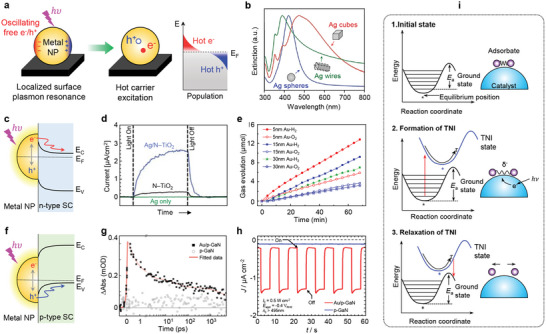
a) Schematic illustration of hot carrier generation in plasmonic metal nanoparticles. Incident photons excite localized surface plasmon resonance (LSPR), inducing the coherent oscillation of free electrons. Non‐radiative decay of the plasmon via Landau damping produces non‐thermal hot electrons and hot holes with broad energy distributions. b) Normalized extinction spectra of Ag nanowires (green), nanospheres (blue), and nanocubes (red). The LSPR peak position varies with nanoparticle shape, allowing tunable absorption of visible light and spectral overlap with catalytic transitions. Reproduced with permission.^[^ ^76^ ^]^ Copyright 2011, Springer Nature. c) Energy band diagram of a plasmonic metal nanoparticle (e.g., Au, Ag) interfaced with an n‐type semiconductor. Hot electrons generated by LSPR excitation can be injected over the Schottky barrier into the conduction band of the semiconductor, enabling photocatalytic reactions or carrier detection. d) Time‐dependent photocurrent measurement of a Ag/TiO_2_ Schottky junction photoanode under visible light illumination (λ = 400–500 nm). The light source was a 300 W Xe arc lamp with an intensity of ≈100 mW cm^−^^2^. Compared to bare Ag or bare TiO_2_, the hybrid structure exhibits significantly higher photocurrent during photoelectrochemical water splitting, attributed to enhanced hot carrier generation and interfacial charge transfer. Reproduced with permission.^[^ ^78^ ^]^ Copyright 2011, American Chemical Society. e) Size‐dependent photocatalytic activity of Au–TiO_2_ hybrid photoelectrodes during water splitting. Smaller Au nanoparticles (e.g., 5 nm) show enhanced H_2_ evolution rate under visible light (λ > 415 nm, intensity ≈80 mW cm^−^^2^, AM 1.5G equivalent), attributed to more efficient hot electron generation and reduced Schottky barrier, as confirmed by conductive AFM and UPS analysis. Reproduced with permission.^[^ ^80^ ^]^ Copyright 2018, The Royal Society of Chemistry. f) Energy band diagram of a plasmonic metal nanoparticle on a p‐type semiconductor. In this configuration, hot holes excited in the metal can overcome the Schottky barrier and be injected into the valence band of the p‐type semiconductor, driving oxidation reactions. g) Ultrafast transient absorption spectroscopy of Au/p‐GaN heterojunctions under 530 nm excitation (pump) and 4.85 µm IR probe. The sharp increase in IR absorption indicates hot‐hole injection into GaN within <200 fs. Hot‐hole transfer was found to influence hot‐electron relaxation kinetics within the metal. Reproduced with permission.^[^ ^81^ ^]^ Copyright 2020, Springer Nature. h) Steady‐state photocurrent response of a plasmonic Au/p‐GaN photocathode under chopped visible light illumination (λ > 495 nm, 100 mW cm^−^^2^). Compared to bare p‐GaN, the hybrid structure exhibits higher photocurrent and improved photoelectrochemical CO_2_ reduction selectivity (CO:H_2_ = 5:1), confirming efficient hot‐hole‐mediated charge separation. Reproduced with permission.^[^ ^83^ ^]^ Copyright 2018, American Chemical Society. i) Mechanistic model of hot‐electron‐mediated bond activation via transient negative ion (TNI) formation. (1) Photon‐excited hot electron is injected into the LUMO of an adsorbate, forming a TNI. (2) The TNI traverses the reaction coordinate, gaining kinetic energy. (3) Electron relaxes back into the metal, leaving the adsorbate vibrationally excited, potentially overcoming the activation barrier. Reproduced with permission.^[^ ^76^ ^]^ Copyright 2011, Springer Nature.

**Figure** **7**a,b describes the physical origin of hot carriers in plasmonic metals. When illuminated with photons resonant with the localized surface plasmon resonance (LSPR), conduction electrons in metals such as Au or Ag undergo collective oscillations. These plasmons can decay nonradiatively via Landau damping, resulting in hot electrons and holes with broad, nonthermal energy distributions.^[^ ^72^, ^73^, ^74^, ^75^ ^]^ The energy spectrum and distribution of these hot carriers are highly sensitive to particle geometry. In particular, extinction spectra shown in Figure 7b illustrate how nanocube, nanosphere, and nanowire morphologies yield distinct LSPR peak positions, thereby providing tunability over the energy range of generated carriers.^[^ ^76^, ^77^ ^]^ This shape‐dependent plasmonic tuning is critical for matching carrier energies to catalytic transitions or Schottky barrier thresholds.

Having described the principles of hot carrier generation, we next consider how these carriers influence catalytic reactions when plasmonic metals are integrated with n‐type semiconductors. One widely studied configuration involves the formation of a Schottky junction between a metal and an n‐type semiconductor, which permits only hot electrons with sufficient energy to overcome the Schottky barrier to be injected into the semiconductor. This energetic filtering process makes the system a valuable platform for correlating interfacial charge transfer with catalytic activity. In Figure 7c, a band diagram depicts this charge transfer pathway, highlighting the injection of photoexcited hot electrons from the metal into the conduction band of the semiconductor. This concept is validated experimentally in Figure 7d by Ingram and Linic,^[^ ^78^, ^79^ ^]^ who constructed Ag/TiO_2_ nanodiodes and observed that under visible illumination, the devices produced significantly enhanced photocurrents during hydrogen evolution compared to bare TiO_2_. These findings demonstrated that plasmonically generated hot electrons are efficiently transferred across the Schottky barrier and actively participate in catalytic proton reduction. Notably, the effectiveness of this process was found to depend on the interfacial barrier height, which is influenced by the size of the metal nanoparticle. As shown in Figure 7e, ultraviolet photoelectron spectroscopic measurements have confirmed that smaller plasmonic nanoparticles lead to reduced barrier heights, thus promoting more efficient electron injection.^[^ ^80^ ^]^ This cascade of energetic alignment, carrier transfer, and reactivity underscores the importance of nanoscale structural control in optimizing plasmon‐assisted catalysis.

In contrast to hot electron‐driven systems, Figure 7f–h describes on catalytic architectures based on p‐type semiconductors, where the injection of hot holes governs the charge transfer dynamics. These systems have attracted growing attention for their capacity to facilitate oxidation reactions and broaden the accessible range of photoelectrochemical transformations. Figure 7f provides a schematic band alignment illustrating how plasmon‐induced hot holes may transfer into the valence band of a p‐type semiconductor. This interfacial process has been probed using ultrafast optical spectroscopy. In Figure 7g, Tagliabue et al. report femtosecond‐resolved transient absorption data for Au/p‐GaN heterostructures, revealing sub‐200 fs hot hole injection events upon 530 nm illumination.^[^ ^81^ ^]^ These time‐resolved experiments demonstrate not only the feasibility of ultrafast hot hole transfer but also its influence on intraband relaxation pathways within the metal, highlighting complex feedback between carrier injection and metal electron dynamics.^[^ ^82^ ^]^

The functional consequences of hot hole injection are reflected in catalytic performance. Figure 7h shows results from DuChene, who investigated CO_2_ electroreduction on Au/p‐GaN photocathodes.^[^ ^83^ ^]^ Upon illumination, not only was the photocurrent enhanced, but the selectivity for CO production over H_2_ also improved significantly, shifting the product ratio from 4:1 to 5:1. These changes were attributed to the role of hot holes in modifying adsorbate‐surface interactions and stabilizing reaction intermediates, thereby demonstrating that hot hole transfer can exert both kinetic and thermodynamic control over catalytic outcomes.

While the prior examples emphasize experimental validation, theoretical perspectives also play a key role in interpreting the influence of hot carriers on surface chemistry. To this end, Figure 7i introduces a mechanistic model based on the transient negative ion (TNI) concept. According to this model, hot electrons transiently occupy the LUMO of a chemisorbed species, forming an anionic intermediate.^[^ ^76^, ^84^, ^85^ ^]^ This transient state enables the adsorbate to move along the reaction coordinate before the electron relaxes back into the metal, leaving the molecule in a vibrationally excited state. This electronically nonadiabatic process has been proposed as a pathway for hot electron‐induced bond activation and has been supported by excited‐state calculations and isotope labeling experiments.^[^ ^37^ ^]^ It provides a compelling explanation for how hot carriers can modulate chemical transformations beyond thermal limits.

Importantly, the systems reviewed in this section are distinct from those based on non‐plasmonic metals such as Pt, Pd, or Ni, where hot carrier generation occurs primarily through interband transitions. These carriers tend to have longer lifetimes but lower energies and are less likely to surmount the Schottky barrier, thereby limiting their effectiveness in interfacial charge transfer. In contrast, the plasmonic systems discussed here generate high‐energy, short‐lived carriers capable of efficient injection and catalytic activation. Moreover, by embedding these plasmonic metals in Schottky junctions, it becomes possible to isolate and study only those carriers that possess sufficient energy for meaningful electronic interaction across the interface. This architectural approach allows for more precise mechanistic interpretation and eliminates ambiguity associated with photothermal effects or broad‐spectrum excitation. Altogether, the studies presented in Figure 7 provide a multidimensional understanding of how photon‐induced hot carriers can drive and regulate catalytic reactions. Through a combination of theoretical modeling, time‐resolved spectroscopy, and operando device measurements, the role of hot electrons and holes is elucidated across a variety of photoelectrochemical systems. These insights not only deepen our mechanistic knowledge but also pave the way for designing nanocatalysts with tunable optoelectronic properties tailored for specific chemical transformations.

### 5.2 Future Perspectives: Nanodevices for Electron‐Driven Modulation of Catalytic Reactions

**Figure 8:**
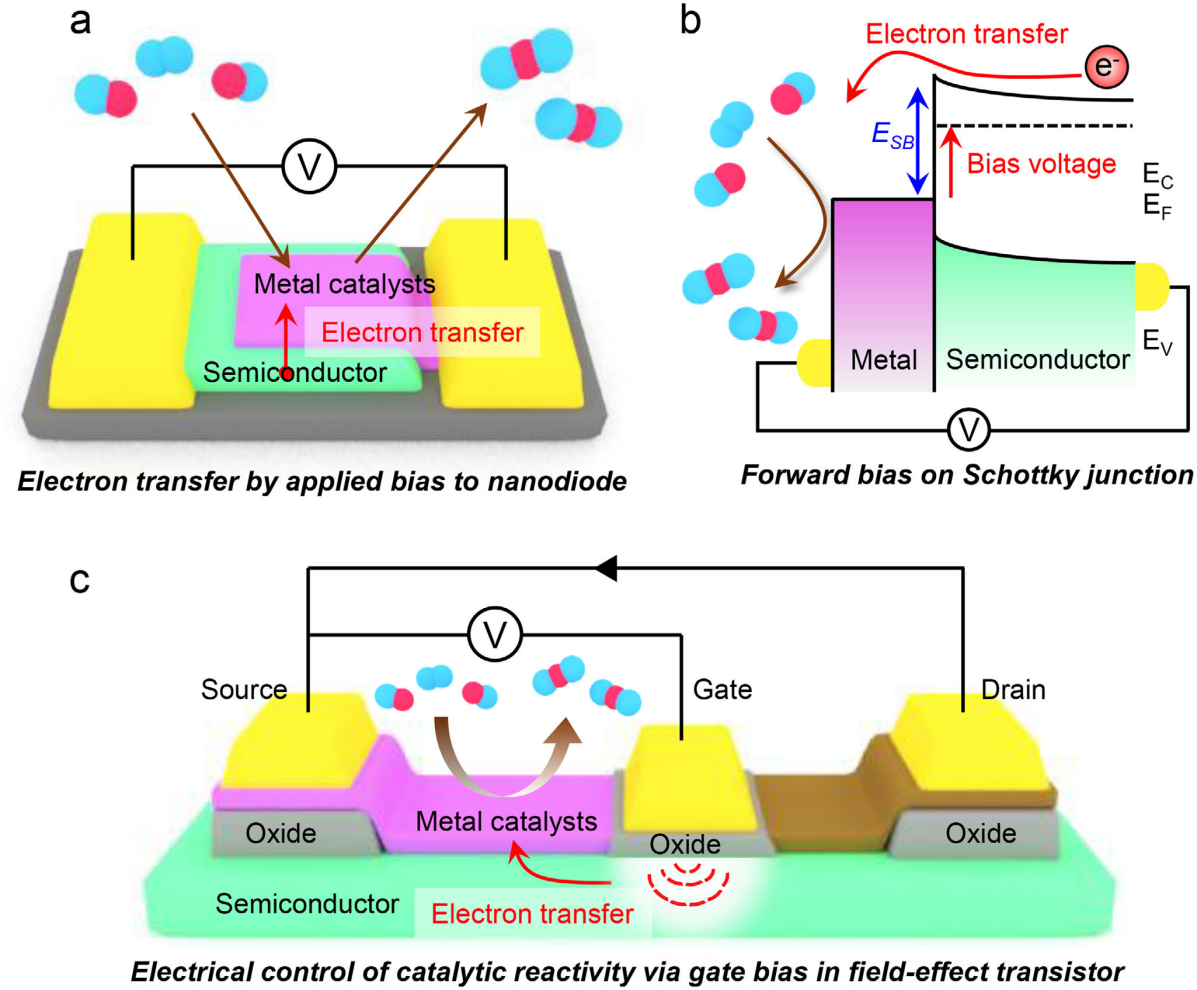
a) Schematic of a nanodiode where a forward bias is applied to the Schottky junction. b) Band diagram of a Schottky junction under forward bias. Electrons from the semiconductor are injected into the metal, modifying the electronic structure of the catalyst. c) Schematic of a FETs structure where the gate bias is applied to modulate electron transfer to the metal catalyst.

The ability to detect hot electron transport in catalytic reactions has provided significant insights into non‐adiabatic energy dissipation pathways. However, a key question remains unanswered: Can we actively control catalytic activity by modulating electron transfer at the metal‐semiconductor interface? Moving beyond passive hot electron detection, the next frontier in catalytic nanodevices focuses on utilizing external bias voltages to manipulate charge transfer and subsequently enhance reaction kinetics. As illustrated in **Figure** **8**, applying a forward bias to a Schottky junction (Figure 8a) injects electrons from the semiconductor into the metal, increasing the electron density on the catalyst surface. This charge accumulation alters the electronic structure of the catalyst, potentially lowering activation barriers and shifting reaction selectivity. The band diagram in Figure 8b further demonstrates how an applied bias modifies the Schottky barrier, facilitating controlled electron transfer to surface‐adsorbed reactants.

To expand the applicability of electron‐driven catalytic modulation beyond model systems such as methanol or hydrogen oxidation, future research must address key challenges associated with more complex and industrially relevant reactions—including photocatalytic methane coupling, dry reforming of methane, hydrogen production via water splitting or the water–gas shift reaction, and selective CO_2_ hydrogenation. These high‐value transformations often involve thermodynamically stable molecules or multi‐electron reaction pathways, necessitating stronger control over charge carrier generation and injection. Recent advances in photon‐driven hot carrier catalysis provide promising strategies for such extensions. For instance, Au–ZnO/TiO_2_ hybrid photocatalysts have demonstrated selective oxidative CH_4_ coupling to C_2_H_6_ under visible light via LSPR‐driven charge separation,^[^ ^86^ ^]^ while Cu‐based plasmonic nanocatalysts enabled low‐temperature photo‐driven water–gas shift reactions through efficient LSPR‐induced hot electron injection.^[^ ^87^, ^88^ ^]^ Moreover, bimetallic nanostructures, such as AuIr composites on InGaN nanowires and RuCo single‐atom alloys, have shown photon‐enhanced activity and selectivity in hydrogenation and syngas conversion.^[^ ^89^, ^90^ ^]^ These examples suggest that catalytic nanodevices, when coupled with tailored light–matter interactions or electrical biasing strategies, could be designed to modulate hot carrier flow and steer challenging reactions toward desired products. Incorporating such methodologies into nanodevice architectures represents a promising path toward broadening the scope of electron‐controlled catalysis.

Moreover, integrating three‐terminal devices, such as field‐effect transistors (FETs), introduces an additional degree of tunability (Figure 8c). In this configuration, the gate voltage modulates charge accumulation in the catalyst, allowing dynamic control over reaction rates. This approach enables a real‐time electronic modulation of catalytic activity, paving the way for a “nanodevice for electron‐driven catalysis”, where electrical stimuli regulate reaction pathways at the molecular level. To validate this concept, future studies should focus on in situ spectroscopic measurements on catalytic nanodevices under applied bias conditions. Real‐time surface analysis will allow direct observation of how electron transfer influences surface adsorbates and reaction intermediates. This will provide crucial insights into the fundamental mechanisms by which applied voltage alters reaction kinetics. Ultimately, this research direction aims to bridge the gap between conventional thermal catalysis and electronic control of reaction dynamics. By harnessing catalytic nanodevices through bias‐induced charge transfer, we can unlock new opportunities for selectivity tuning, energy‐efficient catalysis, and next‐generation electrocatalytic systems.

## 6 Conclusions and Perspectives

This review has outlined the evolution of catalytic nanodevice platforms for detecting hot electrons generated during exothermic catalytic reactions at metal–semiconductor interfaces. Starting from early studies under vacuum conditions using thin‐film model catalysts, the field has progressively advanced toward more complex and practical systems operating under ambient pressures and incorporating nanostructured catalysts. Across these diverse platforms, metal–semiconductor Schottky nanodiodes have emerged as versatile tools for probing non‐adiabatic energy dissipation processes and enabling the real‐time detection of charge carriers generated during catalytic reactions. While much of the existing literature in photoelectrochemistry and plasmonic catalysis has focused on how photon‐excited hot carriers can drive chemical transformations, this review emphasizes a complementary perspective: how chemical reactions themselves—through their intrinsic exothermicity—can excite non‐equilibrium carriers that may be detected as electronic signals. This direction opens new opportunities for understanding transient interfacial processes and exploring the interplay between reaction energetics and electronic excitation at catalytic surfaces.

Looking ahead, the concept of a “Catalytic Nanodevice” extends beyond passive detection of reaction‐induced hot carriers. By integrating catalytic surfaces with semiconductor architectures, such devices could, in the future, enable active control of interfacial charge flows via externally applied bias voltages. This approach, distinct from photon‐induced carrier generation, offers a fundamentally different strategy for investigating and potentially modulating catalytic reactions through electronic control. The continued development of these nanodevice platforms may provide valuable insights into the design of catalytic systems that integrate reaction‐driven and externally controlled charge transfer phenomena. Ultimately, this review highlights the emerging potential of catalytic nanodevices as a versatile platform for probing and modulating reaction‐induced charge transfer processes, paving the way for deeper insights into the dynamic interplay between surface chemistry, electronic excitation, and catalytic performance.

## Conflict of Interest

The authors declare no conflict of interest.

## References

[1] G. A. Somorjai , Y. Li , Introduction to Surface Chemistry and Catalysis, John Wiley & Sons, Hoboken, NJ, USA, 2010.

[2] G. Ertl , Angew. Chem., Int. Ed. 2008, 47, 3524. 10.1002/anie.200800480 18357601

[3] H. J. Freund , G. Meijer , M. Scheffler , R. Schlögl , M. Wolf , Angew. Chem., Int. Ed. 2011, 50, 10064. 10.1002/anie.201101378 21960461

[4] G. A. Somorjai , J. Y. Park , Angew. Chem., Int. Ed. 2008, 47, 9212. 10.1002/anie.200803181 19006127

[5] J. Y. Park , L. R. Baker , G. A. Somorjai , Chem. Rev. 2015, 115, 2781. 25791926 10.1021/cr400311p

[6] C. T. Campbell , Nat. Chem. 2012, 4, 597. 22824888 10.1038/nchem.1412

[7] F. Calaza , C. Stiehler , Y. Fujimori , M. Sterrer , S. Beeg , M. Ruiz‐Oses , N. Nilius , M. Heyde , T. Parviainen , K. Honkala , Angew. Chem., Int. Ed. 2015, 54, 12484. 10.1002/anie.201501420 26012347

[8] M. Bonn , S. Funk , C. Hess , D. N. Denzler , C. Stampfl , M. Scheffler , M. Wolf , G. Ertl , Science 1999, 285, 1042. 10446045 10.1126/science.285.5430.1042

[9] T.‐C. Shen , C. Wang , G. Abeln , J. Tucker , J. W. Lyding , P. Avouris , R. Walkup , Science 1995, 268, 1590. 17754609 10.1126/science.268.5217.1590

[10] B. Hammer , J. K. Nørskov , Adv. Catal. 2000, 45, 71.

[11] S. Linic , U. Aslam , C. Boerigter , M. Morabito , Nat. Mater. 2015, 14, 567. 25990912 10.1038/nmat4281

[12] P. Christopher , H. Xin , S. Linic , Nat. Chem. 2011, 3, 467. 21602862 10.1038/nchem.1032

[13] A. M. Wodtke , Chem. Soc. Rev. 2016, 45, 3641. 27152489 10.1039/c6cs00078a

[14] C. Crowell , W. Spitzer , L. Howarth , E. LaBate , Phys. Rev. 1962, 127, 2006.

[15] S. W. Lee , H. Lee , Y. Park , H. Kim , G. A. Somorjai , J. Y. Park , Surf. Sci. Rep. 2021, 76, 100532.

[16] S. W. Lee , H. Kim , J. Y. Park , Nano Lett. 2023, 23, 5373. 36930862 10.1021/acs.nanolett.3c00173

[17] B. Gergen , H. Nienhaus , W. H. Weinberg , E. W. McFarland , Science 2001, 294, 2521. 11752571 10.1126/science.1066134

[18] H. Nienhaus , B. Gergen , W. Weinberg , E. McFarland , Surf. Sci. 2002, 514, 172. 10.1126/science.1066134 11752571

[19] S. W. Lee , W. Park , H. Lee , H. C. Song , Y. Jung , J. Y. Park , ACS Catal. 2019, 9, 8424.

[20] J. Nørskov , D. Newns , B. Lundqvist , Surf. Sci. 1979, 80, 179.

[21] O. Bünermann , H. Jiang , Y. Dorenkamp , A. Kandratsenka , S. M. Janke , D. J. Auerbach , A. M. Wodtke , Science 2015, 350, 1346. 26612832 10.1126/science.aad4972

[22] J. D. White , J. Chen , D. Matsiev , D. J. Auerbach , A. M. Wodtke , Nature 2005, 433, 503. 15690036 10.1038/nature03213

[23] E. Hasselbrink , Curr. Opin. Solid State Mater. Sci. 2006, 10, 192.

[24] A. M. Wodtke , D. Matsiev , D. J. Auerbach , Prog. Surf. Sci. 2008, 83, 167.

[25] J. C. Tully , Annu. Rev. Phys. Chem. 2000, 51, 153. 11031279 10.1146/annurev.physchem.51.1.153

[26] N. Shenvi , S. Roy , J. C. Tully , Science 2009, 326, 829. 19892977 10.1126/science.1179240

[27] M. Head‐Gordon , J. C. Tully , Phys. Rev. B 1992, 46, 1853. 10.1103/physrevb.46.1853 10003840

[28] B. Kasemo , E. Törnqvist , J. Nørskov , B. Lundqvist , Surf. Sci. 1979, 89, 554.

[29] D. Mantell , S. Ryali , B. Halpern , G. Haller , J.‐B. Fenn , Chem. Phys. Lett. 1981, 81, 185.

[30] M. Born , J. R. Oppenheimer , Ann. Phys. 1927, 389, 457.

[31] T. Greber , Surf. Sci. Rep. 1997, 28, 3.

[32] Y. Huang , C. T. Rettner , D. J. Auerbach , A. M. Wodtke , Science 2000, 290, 111. 11021790 10.1126/science.290.5489.111

[33] G. E. Ewing , Acc. Chem. Res. 1992, 25, 292.

[34] C. Clavero , Nat. Photonics 2014, 8, 95.

[35] E. G. Karpov , I. Nedrygailov , Phys. Rev. B 2010, 81, 205443.

[36] S. W. Lee , J. M. Kim , W. Park , H. Lee , G. R. Lee , Y. Jung , Y. S. Jung , J. Y. Park , Nat. Commun. 2021, 12, 40. 33397946 10.1038/s41467-020-20293-y PMC7782808

[37] H. Nienhaus , Surf. Sci. Rep. 2002, 45, 3.

[38] B. Kasemo , L. Wallden , Surf. Sci. 1975, 53, 393.

[39] A. Böttcher , R. Grobecker , R. Imbeck , A. Morgante , G. Ertl , J. Chem. Phys. 1991, 95, 3756.

[40] T. Greber , K. Freihube , R. Grobecker , A. Böttcher , K. Hermann , G. Ertl , D. Fick , Phys. Rev. B 1994, 50, 8755. 10.1103/physrevb.50.8755 9974896

[41] D. Andersson , B. Kasemo , L. Wallden , Surf. Sci. 1985, 152, 576.

[42] L. Brus , J. Comas , C. Luminescence , J. Chem. Phys. 1971, 54, 2771.

[43] H. Nienhaus , H. Bergh , B. Gergen , A. Majumdar , W. Weinberg , E. McFarland , Phys. Rev. Lett. 1999, 82, 446.

[44] A. M. Wodtke , J. C. Tully , D. J. Auerbach , Int. Rev. Phys. Chem. 2004, 23, 513.

[45] D. Diesing , E. Hasselbrink , Chem. Soc. Rev. 2016, 45, 3747. 27186600 10.1039/c5cs00932d

[46] B. R. Cuenya , H. Nienhaus , E. W. McFarland , Phys. Rev. B 2004, 70, 115322.

[47] X. Liu , B. R. Cuenya , E. W. McFarland , Sens. Actuators, B 2004, 99, 556.

[48] A. Hervier , J. R. Renzas , J. Y. Park , G. A. Somorjai , Nano Lett. 2009, 9, 3930. 19731919 10.1021/nl9023275

[49] H. Lee , I. I. Nedrygailov , C. Lee , G. A. Somorjai , J. Y. Park , Angew. Chem., Int. Ed. 2015, 54, 2340. 10.1002/anie.201410951 25645508

[50] S. W. Lee , B. Jeon , H. Lee , J. Y. Park , J. Phys. Chem. Lett. 2022, 13, 9435. 36194546 10.1021/acs.jpclett.2c02319

[51] I. I. Nedrygailov , J. Y. Park , Chem. Phys. Lett. 2016, 645, 5.

[52] J. Trail , M. Graham , D. Bird , M. Persson , S. Holloway , Phys. Rev. Lett. 2002, 88, 166802. 11955246 10.1103/PhysRevLett.88.166802

[53] K. C. Goddeti , H. Lee , B. Jeon , J. Y. Park , Chem. Commun. 2018, 54, 8968. 10.1039/c8cc04288h 29987273

[54] B. Jeon , H. Lee , K. C. Goddeti , J. Y. Park , ACS Appl. Mater. Interfaces 2019, 11, 15152. 30939872 10.1021/acsami.9b02863

[55] I. I. Nedrygailov , C. Lee , S. Y. Moon , H. Lee , J. Y. Park , Angew. Chem., Int. Ed. 2016, 128, 11017. 10.1002/anie.201603225 27374493

[56] G. A. Somorjai , J. Y. Park , Chem. Soc. Rev. 2008, 37, 2155. 18818818 10.1039/b719148k

[57] J. Y. Park , G. A. Somorjai , ChemPhysChem 2006, 7, 1409. 16739158 10.1002/cphc.200600056

[58] J. R. Renzas , G. A. Somorjai , J. Phys. Chem. C 2010, 114, 17660.

[59] X. Z. Ji , G. A. Somorjai , J. Phys. Chem. B 2005, 109, 22530. 16853934 10.1021/jp054163r

[60] S. N. Maximoff , J. Phys. Chem. C 2017, 121, 2696. 10.1021/acs.jpcb.7b04874 28732447

[61] H. Lee , I. I. Nedrygailov , S. W. Lee , J. Y. Park , Top. Catal. 2018, 61, 915.

[62] S. Yoon , K. Oh , F. Liu , J. H. Seo , G. A. Somorjai , J. H. Lee , K. An , ACS Catal. 2018, 8, 5391.

[63] A. Wittstock , V. Zielasek , J. Biener , C. Friend , M. Bäumer , Science 2010, 327, 319. 20075249 10.1126/science.1183591

[64] G. R. Lee , K. Song , D. Hong , J. An , Y. Roh , M. Kim , D. Kim , Y. S. Jung , J. Y. Park , Nat. Commun. 2025, 16, 2909. 40133268 10.1038/s41467-025-57946-9 PMC11937275

[65] I. I. Nedrygailov , E. G. Karpov , E. Hasselbrink , D. Diesing , J. Vac. Sci. Technol., A 2013, 31, 021101.

[66] J. P. Meyburg , I. I. Nedrygailov , E. Hasselbrink , D. Diesing , Rev. Sci. Instrum. 2014, 85, 104102. 25362420 10.1063/1.4896979

[67] J. R. Creighton , M. E. Coltrin , J. Phys. Chem. C 2012, 116, 1139.

[68] H. Lee , S. Yoon , J. Jo , B. Jeon , T. Hyeon , K. An , J. Y. Park , Faraday Discuss. 2019, 214, 353. 30810549 10.1039/c8fd00136g

[69] E. Lee , B. Jeon , H. Choi , J. Kim , J. Kim , G. Han , K. An , H. Y. Kim , J. Y. Park , S. W. Lee , ACS Catal. 2024, 14, 5520.

[70] S. Yoon , J. Jo , B. Jeon , J. Lee , M. G. Cho , M. H. Oh , B. Jeong , T. J. Shin , H. Y. Jeong , J. Y. Park , ACS Catal. 2021, 11, 1516.

[71] H. Lee , J. Lim , C. Lee , S. Back , K. An , J. W. Shin , R. Ryoo , Y. Jung , J. Y. Park , Nat. Commun. 2018, 9, 5114. 29884825 10.1038/s41467-018-04713-8 PMC5993833

[72] L. Zhou , Q. Huang , Y. Xia , Chem. Rev. 2024, 124, 8597. 38829921 10.1021/acs.chemrev.4c00165 PMC11273350

[73] K. Song , H. Lee , M. Lee , J. Y. Park , ACS Energy Lett. 2021, 6, 1333.

[74] M. J. Kale , T. Avanesian , P. Christopher , ACS Catal. 2014, 4, 116.

[75] L. Zhou , D. F. Swearer , C. Zhang , H. Robatjazi , H. Zhao , L. Henderson , L. Dong , P. Christopher , E. A. Carter , P. Nordlander , Science 2018, 362, 69. 30287657 10.1126/science.aat6967

[76] S. Linic , P. Christopher , D. B. Ingram , Nat. Mater. 2011, 10, 911. 22109608 10.1038/nmat3151

[77] M. W. Knight , H. Sobhani , P. Nordlander , N. J. Halas , Science 2011, 332, 702. 21551059 10.1126/science.1203056

[78] D. B. Ingram , S. Linic , J. Am. Chem. Soc. 2011, 133, 5202. 21425795 10.1021/ja200086g

[79] D. B. Ingram , P. Christopher , J. L. Bauer , S. Linic , ACS Catal. 2011, 1, 1441.

[80] S. Y. Moon , H. C. Song , E. H. Gwag , I. I. Nedrygailov , C. Lee , J. J. Kim , W. H. Doh , J. Y. Park , Nanoscale 2018, 10, 22180. 30484456 10.1039/c8nr05144e

[81] G. Tagliabue , J. S. DuChene , M. Abdellah , A. Habib , D. J. Gosztola , Y. Hattori , W.‐H. Cheng , K. Zheng , S. E. Canton , R. Sundararaman , Nat. Mater. 2020, 19, 1312. 32719510 10.1038/s41563-020-0737-1

[82] A. Manjavacas , J. G. Liu , V. Kulkarni , P. Nordlander , ACS Nano 2014, 8, 7630. 24960573 10.1021/nn502445f

[83] J. S. DuChene , G. Tagliabue , A. J. Welch , W.‐H. Cheng , H. A. Atwater , Nano Lett. 2018, 18, 2545. 29522350 10.1021/acs.nanolett.8b00241

[84] S. Linic , P. Christopher , H. Xin , A. Marimuthu , Acc. Chem. Res. 2013, 46, 1890. 23750539 10.1021/ar3002393

[85] P. Christopher , H. Xin , A. Marimuthu , S. Linic , Nat. Mater. 2012, 11, 1044. 23178296 10.1038/nmat3454

[86] S. Song , H. Song , L. Li , S. Wang , W. Chu , K. Peng , X. Meng , Q. Wang , B. Deng , Q. Liu , Nat. Catal. 2021, 4, 1032.

[87] J. Zhao , Y. Bai , Z. Li , J. Liu , W. Wang , P. Wang , B. Yang , R. Shi , G. I. Waterhouse , X. D. Wen , Angew. Chem., Int. Ed. 2023, 62, 202219299. 10.1002/anie.202219299 36734471

[88] L. Zhou , J. M. P. Martirez , J. Finzel , C. Zhang , D. F. Swearer , S. Tian , H. Robatjazi , M. Lou , L. Dong , L. Henderson , Nat. Energy 2020, 5, 61.

[89] B. Zhou , Y. Ma , P. Ou , Z. Ye , X.‐Y. Li , S. Vanka , T. Ma , H. Sun , P. Wang , P. Zhou , Nat. Catal. 2023, 6, 987.

[90] J. Zhao , J. Liu , Z. Li , K. Wang , R. Shi , P. Wang , Q. Wang , G. I. Waterhouse , X. Wen , T. Zhang , Nat. Commun. 2023, 14, 1909. 37019942 10.1038/s41467-023-37631-5 PMC10076290

